# The HSV-1 mechanisms of cell-to-cell spread and fusion are critically dependent on host PTP1B

**DOI:** 10.1371/journal.ppat.1007054

**Published:** 2018-05-09

**Authors:** Jillian C. Carmichael, Hiroki Yokota, Rebecca C. Craven, Anthony Schmitt, John W. Wills

**Affiliations:** 1 Department of Microbiology and Immunology, Pennsylvania State University, College of Medicine, Hershey, Pennsylvania, United States of America; 2 Department of Biomedical Engineering, Indiana University-Purdue University Indianapolis, Indianapolis, Indiana, United States of America; 3 Department of Veterinary and Biomedical Sciences, Pennsylvania State University, University Park, Pennsylvania, United States of America; Blumburg Institute, UNITED STATES

## Abstract

All herpesviruses have mechanisms for passing through cell junctions, which exclude neutralizing antibodies and offer a clear path to neighboring, uninfected cells. In the case of herpes simplex virus type 1 (HSV-1), direct cell-to-cell transmission takes place between epithelial cells and sensory neurons, where latency is established. The spreading mechanism is poorly understood, but mutations in four different HSV-1 genes can dysregulate it, causing neighboring cells to fuse to produce syncytia. Because the host proteins involved are largely unknown (other than the virus entry receptor), we were intrigued by an earlier discovery that cells infected with wild-type HSV-1 will form syncytia when treated with salubrinal. A biotinylated derivative of this drug was used to pull down cellular complexes, which were analyzed by mass spectrometry. One candidate was a protein tyrosine phosphatase (PTP1B), and although it ultimately proved not to be the target of salubrinal, it was found to be critical for the mechanism of cell-to-cell spread. In particular, a highly specific inhibitor of PTP1B (CAS 765317-72-4) blocked salubrinal-induced fusion, and by itself resulted in a dramatic reduction in the ability of HSV-1 to spread in the presence of neutralizing antibodies. The importance of this phosphatase was confirmed in the absence of drugs by using PTP1B^-/-^ cells. Importantly, replication assays showed that virus titers were unaffected when PTP1B was inhibited or absent. Only cell-to-cell spread was altered. We also examined the effects of salubrinal and the PTP1B inhibitor on the four Syn mutants of HSV-1, and strikingly different responses were found. That is, both drugs individually enhanced fusion for some mutants and reduced fusion for others. PTP1B is the first host factor identified to be specifically required for cell-to-cell spread, and it may be a therapeutic target for preventing HSV-1 reactivation disease.

## Author summary

It is estimated that 67% of the global population is infected with herpes simplex virus type 1 (HSV-1). This virus resides in sensory neurons in a quiescent state but periodically reactivates, producing virus particles that travel down the axon to infect epithelial cells of the skin, where it can be transmitted to additional people. To avoid neutralizing antibodies, herpesviruses have evolved mechanisms for moving directly from one cell to another through their sites of intimate contact; however, the mechanism of cell-to-cell spread is poorly understood. Studies of HSV-1 mutants have implicated numerous viral proteins, but the necessary cellular factors are unknown except for the one that the virus uses to enter cells. Our experiments have identified a cellular enzyme (PTP1B, a tyrosine phosphatase) that is dispensable for the production of infectious virions but is critically important for the cell-to-cell spreading mechanism. Promising drugs targeting PTP1B have already been tested in early clinical trials for possible treatment of obesity and type-2 diabetes, and thus, our study may have immediate utility for attenuating HSV-1 reactivation disease in immunocompromised patients.

## Introduction

There are two ways that viruses can spread to uninfected cells. Cell-free spread occurs when virions are released from an infected cell into their surrounding environment prior to entering a new cell. This, of course, is how viruses spread to new hosts and often between cells within a host. However, some viruses, including all the herpesviruses, also have a “cell-to-cell” spreading mechanism by which virions pass directly through cell junctions, enabling protection from neutralizing antibodies [1, 2]. For example, herpes simplex virus type 1 (HSV-1) utilizes cell-to-cell spread to move directly from mucosal epithelial cells, the initial site of infection, into nearby sensory neurons, where the virus establishes a latent infection. When the virus reactivates, newly formed viral particles travel back down the axon, and cell-to-cell spread is used again to allow passage of the virions into the mucosal epithelium [3, 4]. Importantly, replication-competent mutants of HSV-1 that are defective for cell-to-cell spread fail to infect neurons when tested in animal models, and therefore cannot establish latency [5, 6]. Despite its importance, the mechanism of cell-to-cell spread remains poorly understood for all herpesviruses.

Cell-to-cell spread can be assessed *in vitro* by measuring the sizes of plaques produced in the presence of neutralizing antibodies. These antibodies will inactivate virions released into the medium, preventing cell-free spread. In the presence of neutralizing antibodies, wild type HSV-1 forms large plaques in cell cultures due to its capacity for cell-to-cell spread [7]. In contrast, replication-competent mutants defective for this spreading mechanism exhibit greatly reduced plaque sizes under these conditions [5, 7]. Hence, this assay has been used to identify viral factors involved in cell-to-cell spread.

**Fig 1:**
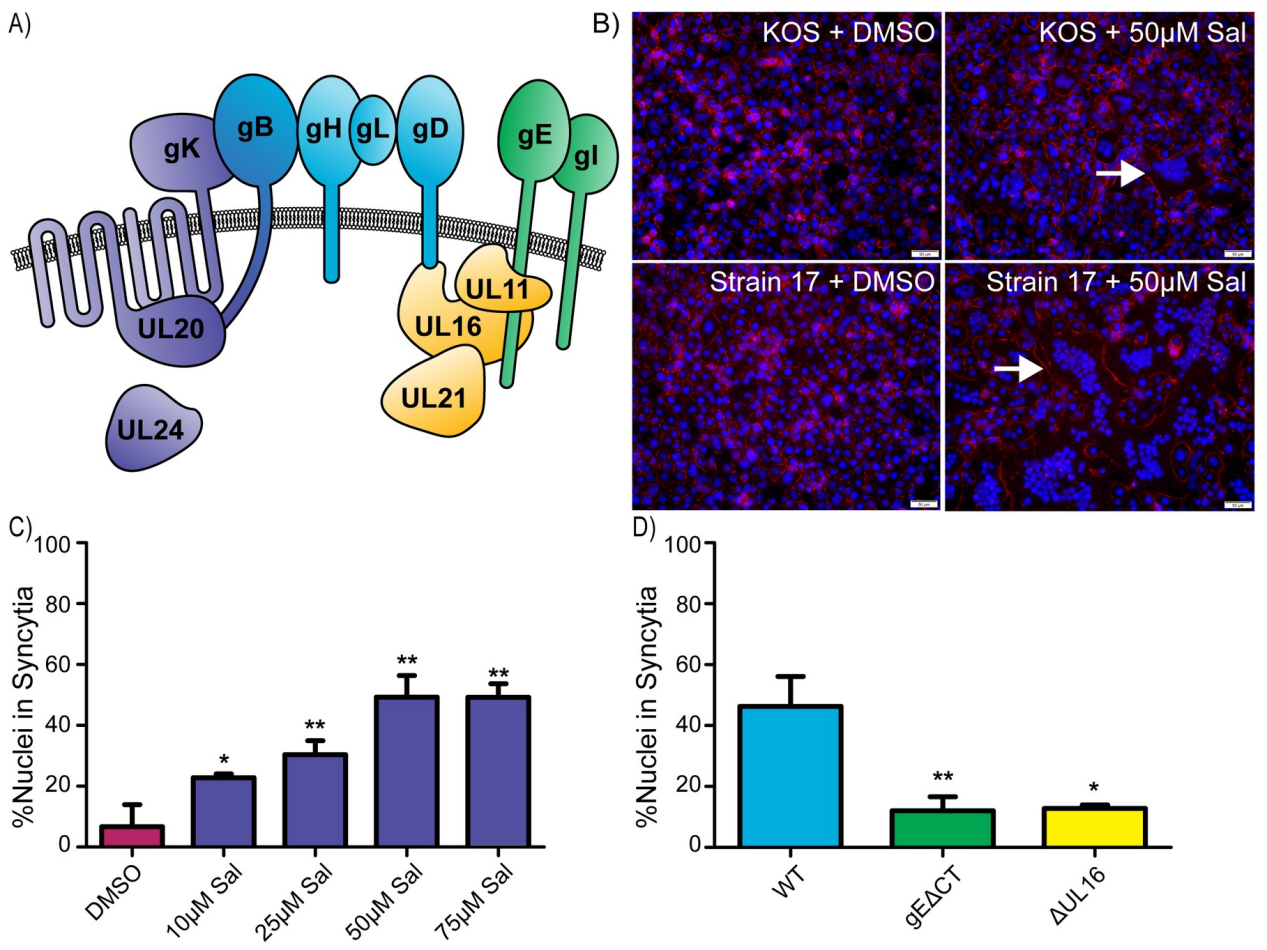
Salubrinal-induced fusion of HSV-infected cells is dependent on accessory proteins. **(A)** Diagram of relevant HSV-1 proteins involved in cell-to-cell spread and syncytia formation. The core fusion proteins are blue (gB, gH/gL, and gD), and proteins that can be altered to create syncytial variants are purple (gK, UL20, UL24, and gB). Two of the accessory glycoproteins are green (gE and gI), and three of the accessory tegument proteins are yellow (UL11, UL16, and UL21). **(B)** Vero cells were infected (MOI = 1) with HSV-1 strains KOS or 17 and incubated in medium containing DMSO or 50 μM salubrinal. At 12 hpi, the cells were immunostained for ZO-1 (red), and nuclei were stained with DAPI. Examples of syncytia are indicated (arrows). **(C)** Cells were infected with the KOS strain (MOI = 0.5) and incubated in the presence of salubrinal, as indicated. At 18 hpi, the cells were immunostained for ZO-1, and DAPI-stained nuclei were scored as being inside syncytia or within single cells. 1000 nuclei were scored per image for 3 replicates. Data are represented as mean ±SD, and statistical significance was determined by a student T-test. **(D)** Cells were infected (MOI = 0.5) with WT, gEΔCT, or ΔUL16 viruses and incubated in the presence of 50 μM salubrinal for 18 hours. DAPI-stained nuclei were scored as in (1C).

The complexity of cell-to-cell spread for HSV-1 is reflected in the many viral proteins that seem to be required. At the core, four glycoproteins—gB, gH/gL, and gD—form the fusion complex (Fig 1A), which is sufficient for virus entry but not for the cell-to-cell spread mechanism [8]. During entry, gD binds to cellular receptors and transmits a signal through the gH/gL heterodimer to the viral fusion protein, gB [9, 10]. This induces a conformational change in gB, triggering the fusion of the viral envelope with the cell membrane [11]. When genes encoding gD, gH/gL, and gB are co-transfected into cells that express receptors, gB activation also occurs, causing massive fusion of the cells with one another [12]. Since cell fusion rarely occurs in HSV-1 infections, it is clear that additional viral proteins regulate the fusion activity of the core fusion complex.

Two viral multi-pass membrane proteins known to interact with gB are gK and UL20 (Fig 1A). These proteins also interact with each other and function in cell-to-cell spread *in vitro* [13, 14]. gK also regulates the core fusion complex as it blocks cell fusion when co-expressed with gD, gH/gL, and gB [15], and in mouse models, gK-deletion mutants fail to spread into neurons after ocular infection [16]. gE also plays a critical role in cell-to-cell spread, as shown by gE-deletion mutants, which exhibit spreading defects *in vitro* in the presence of neutralizing antibodies and fail to spread into neurons in mouse models [5, 17]. Furthermore, the tegument proteins UL16 and UL21 and peripheral membrane-binding protein UL11 form a complex on the cytoplasmic tail of gE (Fig 1A). In cell cultures, this complex seems to be important for cell-to-cell spread [7, 18]. Other HSV-1 proteins that have been implicated in cell-to-cell spread include UL51, gI, and UL34 [19–21].

Strikingly, many of the accessory proteins needed for cell-to-cell spread are also necessary for the syncytial (Syn) phenotype exhibited by certain HSV-1 mutants. These mutants inappropriately cause cell fusion, resulting in large multinucleated cells. Most syncytial mutations (Fig 1A) cause changes in the cytoplasmic tail of gB or the amino-terminal segment of gK, but alterations in UL20 and UL24 can also produce the Syn phenotype [22]. The Syn phenotype requires the core-fusion complex and a variety of accessory proteins. This has been explored in greatest depth for gBsyn mutants, where removal of gE, UL16, UL11, or UL21 results in loss of the Syn phenotype even though the viruses still replicate [18]. Because their requirements clearly overlap, studies of the Syn mutants of HSV-1 have the potential to reveal mechanistic insights for cell-to-cell spread.

Although much is known about the viral machinery involved in cell-to-cell spread, virtually nothing is known about required host factors, other than the need for gD to interact with a receptor [23]. A potential way forward emerged 10 years ago with a report showing that wild-type HSV-1 will cause cells to fuse into massive syncytia when they are treated with the drug salubrinal [24]. We reasoned that the cellular target of salubrinal may be involved in regulating both syncytia formation and cell-to-cell spread.

Salubrinal is well known to prolong the survival of cells experiencing ER stress, which arises when proteins are made faster than they can be folded [25]. The ER stress response, also called the unfolded protein response (UPR), causes the phosphorylation of translation initiation factor eIF2α, which slows translation and allows time for recovery. Later, GADD34 binds to protein phosphatase 1 (PP1) and directs it to eIF2α to remove the inhibitory phosphate [26]. Salubrinal blocks this step, prolonging the stress response, but its cellular target is unknown [27].

Infection with HSV-1 also triggers the ER stress response, but to keep translation going, the virus encodes ICP34.5, a GADD34 homologue, to recruit PP1 and dephosphorylate eIF2α [28]. Salubrinal blocks this step, too, which extends the translation block, thereby reducing the production of infectious virions [25]. For unknown reasons, salubrinal treatment also causes widespread fusion of HSV-1 infected cells, even in the absence of a Syn mutation or ICP 34.5 [24].

We began this study of salubrinal-induced fusion of HSV-1-infected cells with hopes of discovering a host factor involved in the biologically and clinically important process of cell-to-cell spread. In this study, we show that PTP1B, a host tyrosine phosphatase, though not a target of salubrinal, is critical for this type of spread.

## Results

### Salubrinal-induced fusion is dependent on HSV-1 accessory proteins

To confirm that salubrinal stimulates fusion, Vero cells were infected with the wild-type KOS strain of HSV-1 and incubated with increasing concentrations of the drug. The cells were fixed at 18 hours post infection, and the nuclei were stained with DAPI while a tight junction protein, ZO-1, was stained with a fluorescent antibody to help identify the plasma membrane. Only HSV-infected cells treated with salubrinal formed syncytia (Fig 1B). Manual counting of all nuclei within syncytia (defined as 3 or more nuclei per cell) versus all cells with single nuclei revealed a dramatic increase in fusion as the drug concentration increased, reaching a plateau at 50 μM (Fig 1C). As expected, uninfected cells did not exhibit an increase in fusion in response to the drug [24], and the level of fusion present among HSV-infected cells treated with an equal concentration of DMSO matched that of untreated infected cells. Fusion of salubrinal-treated, KOS-infected cells reached ~50% (Fig 1C), and this was even higher for strain 17-infected cells (Fig 1B).

A potential explanation for how salubrinal stimulates cell fusion was that its action disrupts protein complexes to allow the core fusion machinery (gD, gH/gL, and gB) to act independently from viral regulatory proteins (Fig 1A). To rule out this possibility, we used viral mutants ΔUL16 and gEΔCT, where UL16 and the cytoplasmic tail of gE were deleted, to examine their effect on salubrinal-induced cell fusion [18, 29]. Neither mutant was able to induce cell fusion with salubrinal treatment (Fig 1D). Thus, it appears that accessory proteins work together with the core fusion machinery during salubrinal-induced fusion.

### Flow cytometry-based method to measure cell fusion

Quantitation of syncytia can be accomplished by manually scoring nuclei (Fig 1C and 1D); however, this is time consuming and limits the number of samples that can be analyzed. Previously, an alternative method for quantitating cell fusion made use of a Coulter counter to measure the disappearance of single-nucleated cells as more cells fuse over time [30]. Since flow cytometry offers greater analytic power than a Coulter counter, we attempted to use it for rapid, quantitative measurements.

**Fig 2:**
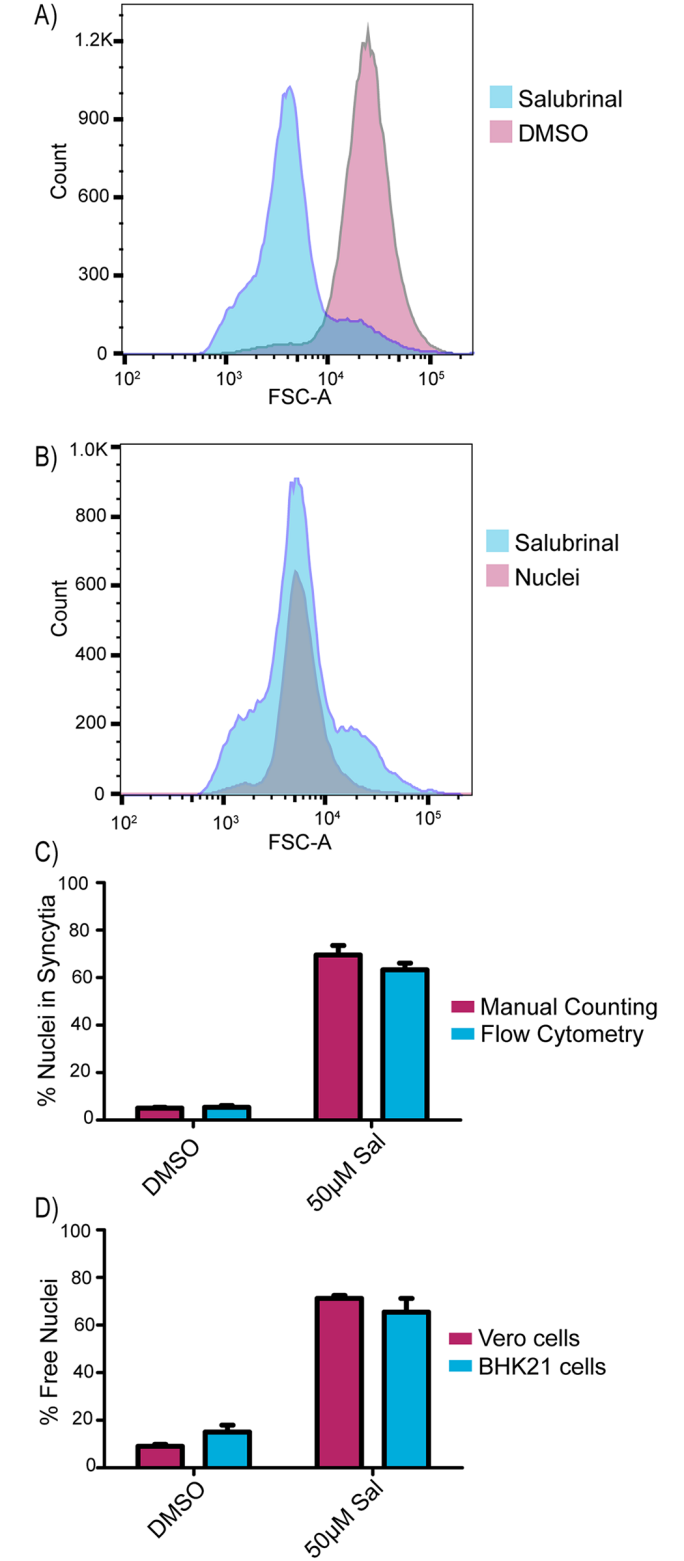
Flow cytometry-based method to measure cell fusion. **(A)** Vero cells were infected with strain 17 (MOI = 3) and incubated in media containing DMSO or 50 μM salubrinal. At 12 hpi, the samples were trypsinized, harvested in 2% PFA, and stained with PI to label nuclei. Samples were run through a cell analyzer and PI-positive events were gated. **(B)** Cells were infected with strain 17 (MOI = 3) and incubated for 12 hours in medium containing DMSO or 50 μM salubrinal. The salubrinal sample was processed as in (2A), and the DMSO-treated cells were lysed with buffer containing Triton X-100 to release all the nuclei, which were stained with PI. **(C)** Cells were infected with strain 17 (MOI = 3) and treated with DMSO or 50 μM salubrinal in duplicate. At 12 hpi, one replicate (blue bars) was harvested and processed for flow cytometry as in (2A), and the other (pink bars) was immunostained for ZO-1, and DAPI-stained nuclei in syncytia were manually scored as in (1C). The averages of two independent experiments are shown. **(D)** Vero or BHK21 cells were infected (MOI = 3) with strain 17 and treated with DMSO or 50 μM salubrinal. At 12 hpi, the cells were harvested and analyzed by flow cytometry as in (2A).

Strain 17-infected Vero cells were treated with salubrinal or DMSO, and when extensive fusion was visually evident (12 hr post infection), the cells were exposed to trypsin for 5 minutes and subsequently harvested by vigorous pipetting in a solution of 2% paraformaldehyde to generate a homogenous suspension. The samples were then exposed to propidium iodide (PI), which was able to stain all the nuclei since the cells were fixed. The suspensions were analyzed with a flow cytometer to measure particle numbers and sizes (by forward light scattering, FSC-A), and the resulting dot plots (S1 Fig) were converted into histograms (Fig 2A). In the DMSO control, one peak was observed representing the size distribution of intact, single cells present in the suspension (Fig 2A). In contrast, a dramatically smaller-size population representing 75–80% of the PI-positive events was found for the salubrinal-treated sample (Fig 2A). We hypothesized that these smaller particles were nuclei released from syncytia during sample preparation.

To test whether the salubrinal-induced, PI-stained population behaved like free nuclei, a parallel culture of non-fused, HSV-infected cells were harvested and treated with 1% Triton X-100 to remove cell membranes; thereby releasing all the nuclei. These were pelleted, resuspended, and stained with PI prior to flow cytometry analysis. The detergent-released nuclei and most of the particles from salubrinal-induced syncytia were found to be of the same sizes, indicating that free nuclei indeed represent the majority of particles released from the sample of fused cells (Fig 2B). To verify these results accurately reflect the number of nuclei contained in syncytia, duplicate cultures were infected with strain 17 and treated with salubrinal. At 12 hr post infection, one set of cells was fixed and manually analyzed, and the other was harvested, processed via flow cytometry, and the percentage of free nuclei was calculated. Both methods delivered the same results (Fig 2C).

To ascertain whether flow cytometry could be used to analyze fusion in another cell line, strain 17-infected BHK21 cells were treated with salubrinal. Massive cell fusion was apparent upon visual examination, and flow cytometry analysis revealed free nuclei levels of 75–80%, similar to what was seen with salubrinal-induced fusion in Vero cells (Fig 2D). In contrast, transfected C10 cells (used below) were not amenable to flow cytometry because of clumping issues.

### Parameters influencing salubrinal-induced fusion

**Fig 3:**
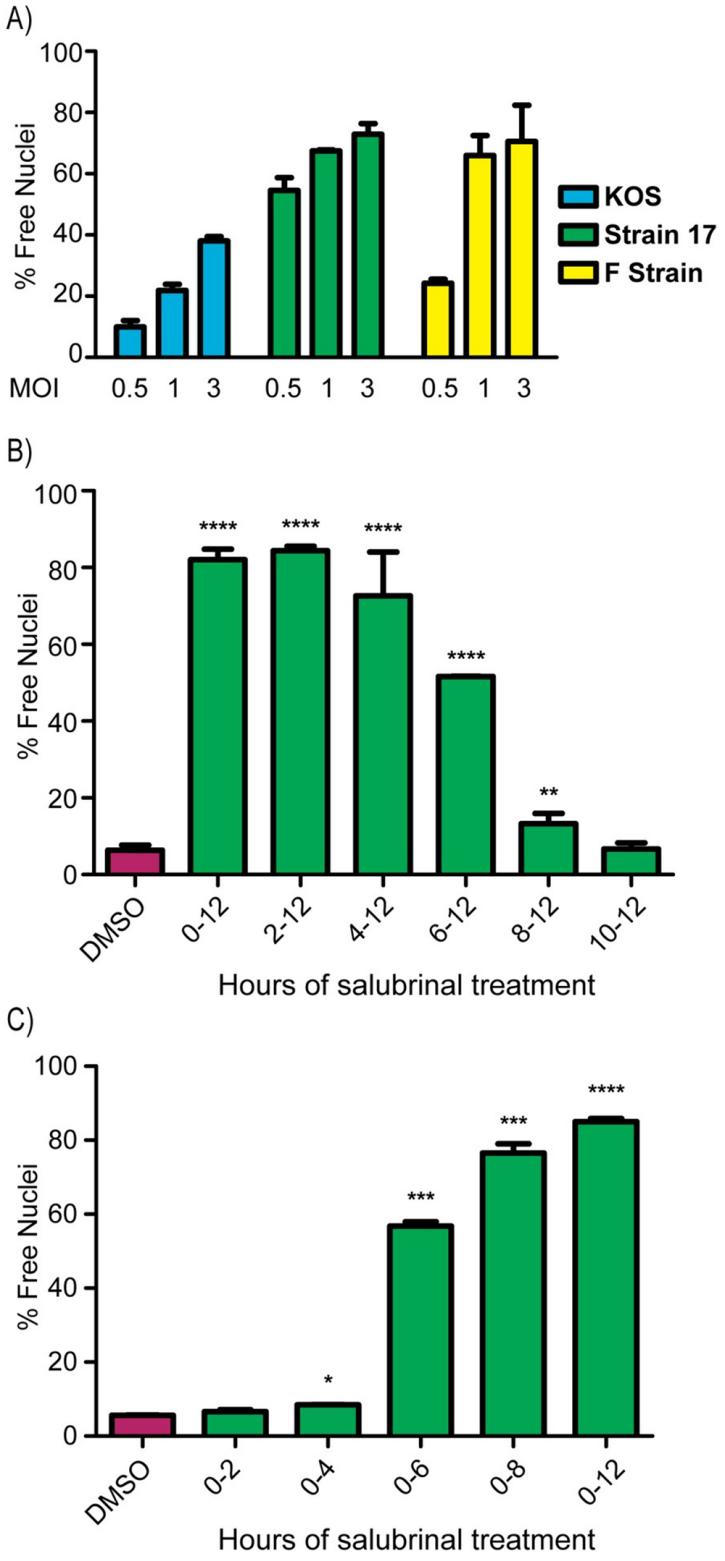
Parameters influencing salubrinal-induced fusion. **(A)** Vero cells were infected with strains KOS, F, or 17 at MOIs of 0.5, 1, or 3 and were incubated in medium containing 50 μM salubrinal. At 12 hpi, the fusion ratio (% free nuclei) was determined by flow cytometry. The mean values (±SD) from 3 independent experiments are plotted. **(B)** After infection with strain 17 (MOI = 3), cells were incubated in medium containing DMSO, and 50 μM salubrinal was added for the indicated time intervals. After a total of 12 hpi, the fusion ratios were measured by flow cytometry. Data are from 2 independent experiments. A student T-test was used to determine statistical significance for samples compared to the DMSO control. **(C)** After infection with strain 17 (MOI = 3), cells were incubated for the indicated time periods in medium containing 50 μM salubrinal, which was then replaced with control medium. At 12 hpi, the fusion ratio was determined by flow cytometry, and the data were analyzed as in (3B).

With a rapid fusion assay in hand, we confirmed that strains 17 and F are more responsive to 50 μM salubrinal than KOS (Fig 3A). Unfortunately, there are too many genetic differences to explain why KOS is somewhat limited in salubrinal-induced fusion [31]. For all three strains, we found that the extent of fusion increased with the multiplicity of infection (MOI), suggesting that infected cells prefer to fuse with other infected cells (Fig 3A). This is reminiscent of cells infected with gKsyn mutants, which prefer to fuse with other infected cells [30] in contrast to gBsyn mutants which have optimal fusion at lower MOIs. Beyond HSV-1, cells infected with wild-type pseudorabies virus (PRV, a porcine alphaherpesvirus) were also stimulated to fuse with salubrinal to similar levels as the HSV-1 KOS strain, but this virus was not investigated any further.

To determine the optimal timing of salubrinal treatment, two distinct time course experiments were conducted. In the first, cells infected with strain 17 at a high MOI received salubrinal at various times post infection, and the cultures were assayed for fusion at 12 hr post infection. This showed that salubrinal could be added as late as 4 hr with no reduction of fusion (Fig 3B). When salubrinal was added at 6 hr, fusion levels dropped to 50%, and no fusion was observed when treatment began at 8 or 10 hr, since by that point, cells were rounding up due to extensive CPE and could no longer fuse. In a reciprocal experiment, salubrinal was added to cells immediately following strain 17 infection and then removed at various times. No fusion was observed when salubrinal was removed prior to 4 hr post infection (Fig 3C); however, total cell fusion reached 50% when salubrinal was removed at 6 hr, and maximal fusion was observed when salubrinal was removed after 8 hr. These data indicate that salubrinal exerts its effect only when the virus enters the late stage of infection, when the structural proteins of the virus begin to be synthesized [32].

### Host protein tyrosine phosphatase 1B is important for salubrinal-induced fusion

**Fig 4:**
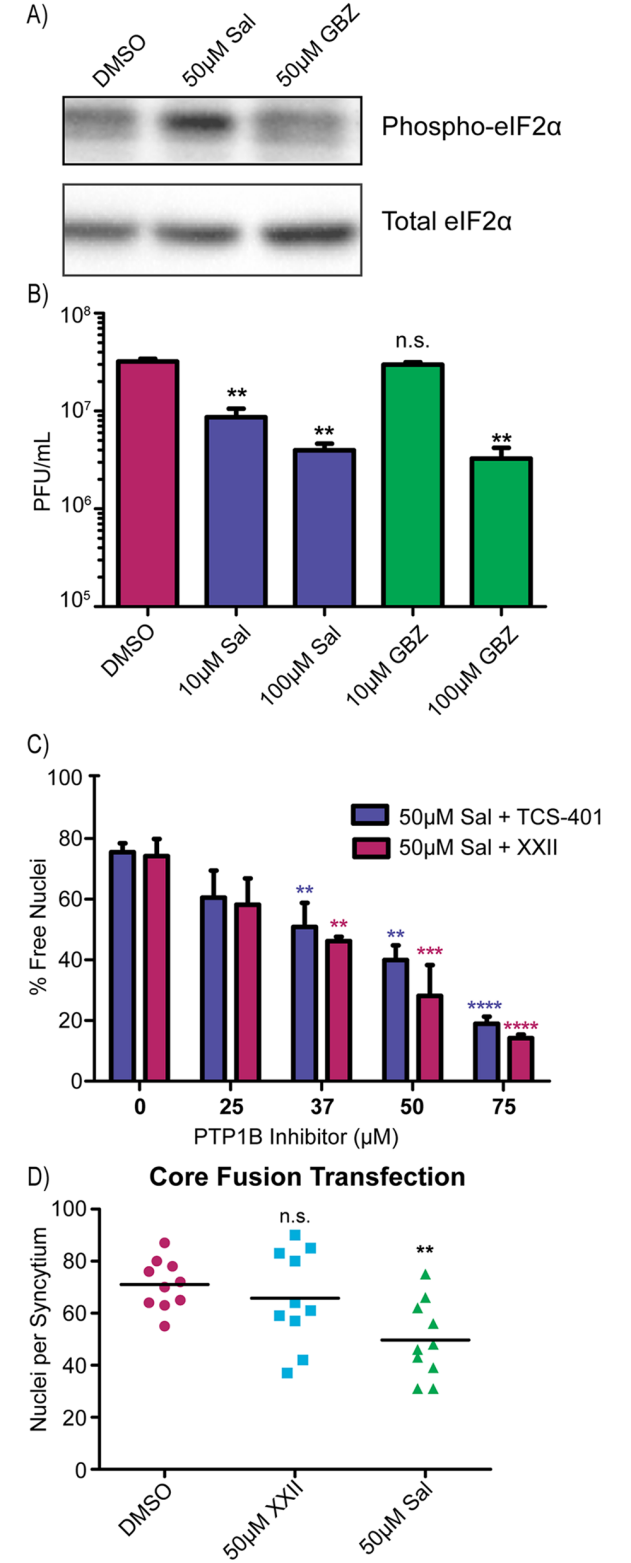
Host protein tyrosine phosphatase 1B is important for salubrinal-induced fusion. **(A)** Vero cells were infected with strain 17 (MOI = 3) and incubated in DMSO, 50 μM salubrinal, or 50 μM guanabenz (GBZ) for 12 hours. Cell lysates were prepared in the presence of phosphatase inhibitors and probed via western blotting for total eIF2α and for phosphorylated eIF2α. **(B)** Cells were infected with KOS (MOI = 3) and incubated in DMSO, salubrinal, or guanabenz. At 24 hpi, cell lysates and media were harvested, and viral titers (cell lysates + media) were measured. Data are the averages of 2 replicates with statistical significance determined by a student T-test. **(C)** Cells were infected with strain 17 (MOI = 3) and treated with 50 μM salubrinal and increasing amounts of PTP1B inhibitors TCS-401 or XXII. At 12 hpi, the fusion ratio was determined by flow cytometry. Data are represented as the mean ±SD from 3 independent experiments, and a student T-test was used to determine statistical significance for samples compared to the salubrinal-only control. **(D)** C10 cells were transfected with plasmids encoding gB, gD, gH, and gL in a 3:1:1:1 ratio and treated with DMSO, 50 μM salubrinal, or 50 μM inhibitor XXII for 16 hours. The 10 largest syncytia per sample were measured by counting the number of nuclei per syncytium. A student T-test was used to compare samples to the DMSO-only control.

Salubrinal treatment is well known to inhibit protein synthesis by increasing eIF2α phosphorylation levels in HSV-infected cells relative to a DMSO control, and this was confirmed in our studies (Fig 4A). It has sometimes been assumed that salubrinal prolongs eIF2α phosphorylation by binding to GADD34 (and the HSV-1 homolog, ICP34.5), thereby preventing it from associating with PP1. However, recent studies emphasize that salubrinal does not, in fact, bind directly to GADD34 [27, 33]. In contrast, the drug guanabenz does act on GADD34 [27, 34], and to test whether it can stimulate fusion, KOS or strain 17-infected cells were incubated with various concentrations. No cell fusion was observed, even at the highest drug concentration tested (100 μM). Moreover, no increase in phosphorylation of eIF2α was observed (Fig 4A), unlike what was found in previous studies of uninfected cells treated with guanabenz [34]. However, both salubrinal and guanabenz exhibited antiviral activity with titers being reduced by 1 log in KOS-infected cells (Fig 4B). These results emphasize the importance of finding the host protein that binds salubrinal so that the mechanism by which it induces fusion can be understood.

In an attempt to identify the target of salubrinal, a biotinylated chemical analogue named UTX-102 was synthesized (S2A Fig). Importantly, this drug displayed similar activity to salubrinal in an established killing assay [35] with MDA-MB-231 breast cancer cells (S2B Fig). These cells were treated with UTX-102, cell lysate was harvested, and avidin agarose beads were used to pull-down protein complexes. Among the proteins identified by LC-MS/MS was protein tyrosine phosphatase 1B (PTP1B), and immunoblot analysis verified that it was in the complex pulled down with UTX-102 (S2C Fig).

PTP1B is an ER-bound tyrosine phosphatase known to modulate a number of different signaling pathways, including events at the plasma membrane [36]. If inhibition of PTP1B in HSV-infected cells is what triggers cell fusion, then treatment with other known inhibitors of this enzyme should do the same. To test this, cells were infected with strain 17 and treated with two different allosteric inhibitors of PTP1B: TCS-401 or inhibitor XXII (CAS 765317-72-4) [37, 38]. Both drugs failed to stimulate fusion.

Because the cellular functions of PTP1B are known to be regulated (e.g., by phosphorylation; [36]), we next considered the possibility that salubrinal might alter substrate specificity of this enzyme. If this were the case, then PTP1B inhibitors might block salubrinal-induced fusion. Accordingly, strain 17-infected cells were incubated with a combination of salubrinal and increasing amounts of each PTP1B inhibitor. In both cases, salubrinal-induced fusion was dramatically decreased with fusion being almost completely ablated at the highest concentrations of PTP1B inhibitors (Fig 4C). While this suggests that PTP1B is critical for the fusion-inducing activity of salubrinal, it does not prove that this host factor is the target of salubrinal. Indeed, other results (see below) suggest that PTP1B is not the target. As inhibitor XXII more effectively limited fusion at lower dosages than the other drug, it was used in the subsequent experiments.

Since all HSV-1 fusion events require the core fusion machinery (gD, gH/gL, and gB), we next asked whether inhibitor XXII would block their activity. Transfected C10 cells, which overexpress the nectin-1 receptor for gD, fused efficiently when the four fusion proteins are co-expressed (S3 Fig, top row), and manual counting revealed an average of 70 nuclei per syncytium (Fig 4D, DMSO control). This did not change when inhibitor XXII was present (Fig 4D and S3 Fig, bottom row). Transfected cells were also treated with salubrinal, and while there was slightly less fusion at 16 hrs post transfection (Fig 4D), by 24 hr the level reached, but did not exceed, that of the control (S3 Fig, middle row). Clearly, PTP1B and the target of salubrinal do not regulate the core fusion machinery in isolation but only when it is in a complex with accessory proteins (Fig 1A).

### Effects of salubrinal and inhibitor XXII on a paramyxovirus

To get a glimpse at whether the effects of salubrinal and inhibitor XXII are limited to herpesviruses, cells infected with PIV5, a paramyxovirus, were treated with the drugs. Neither salubrinal (S4A Fig) nor inhibitor XXII (S4C Fig) stimulated cell fusion. However, experiments with a fusogenic variant, rPIV5-NPΔ4v6 [39], yielded different results. At 3 days post infection, multiple large syncytia were apparent in the DMSO controls, but to our surprise, salubrinal treatment actually reduced cell fusion (S4B Fig). A reduction in the number and sizes of rPIV5-NPΔ4v6 syncytia was also seen with inhibitor XXII (S4D Fig), which is in line with the ability of this inhibitor to block salubrinal-induced fusion of HSV-1. Further investigation of paramyxoviruses is warranted, but we next examined the effects of these two drugs on HSV-1 Syn mutants.

### Differential effects of salubrinal and inhibitor XXII on HSV-1 syncytial variants

**Fig 5:**
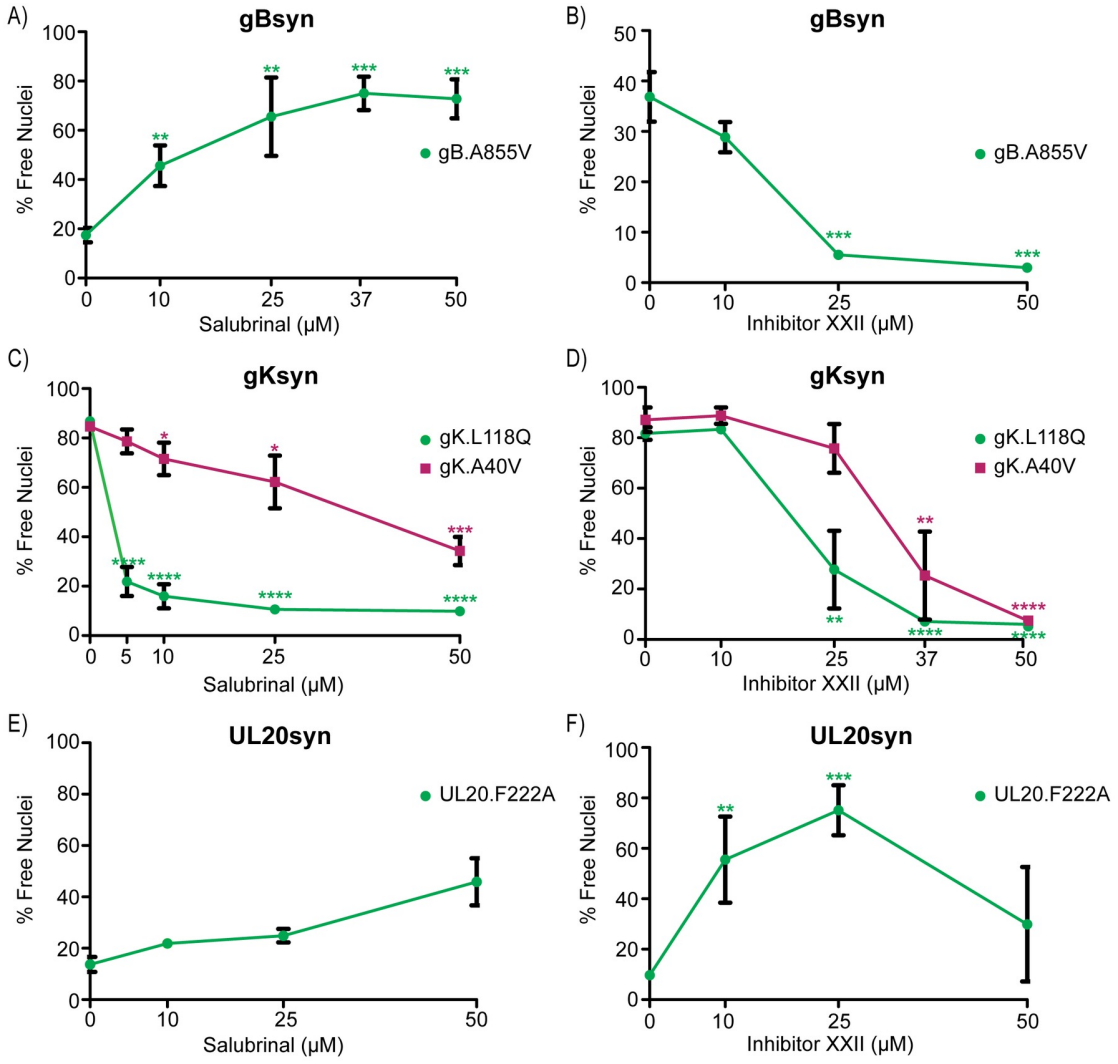
Differential effects of salubrinal and inhibitor XXII on HSV-1 syncytial variants. **(A and B)** Vero cells were infected with mutant gB.A855V at MOIs of (A) 1 or (B) 0.1. Since this mutant prefers to drive fusion with neighboring uninfected cells, the baseline level is higher at the lower MOI. Cells were treated with increasing amounts of (A) salubrinal or (B) inhibitor XXII, and at 12 hpi (A) or 24 hpi (B) the fusion ratios were determined by flow cytometry. The data are represented as the mean ±SD from 3 independent experiments, and a student T-test was used to determine statistical significance for samples compared to the DMSO control. **(C and D)** Cells were infected (MOI = 1) with mutants gK.L118Q or gK.A40V and treated with (C) salubrinal or (D) inhibitor XXII. At 12 hpi, syncytia were analyzed as in (5A). **(E and F)** Cells were infected (MOI = 1) with mutant UL20.F222A and treated with (E) salubrinal or (F) inhibitor XXII. At 12hpi, syncytia were analyzed as in (5A).

Syn variants arise from alterations to any of four different proteins (gB, gK, UL20, or UL24; Fig 1A), and these changes dysregulate the cell-to-cell spreading machinery in ways that are not understood, but are likely at distinct steps. We previously constructed examples of Syn mutants within the KOS strain [7, 18], and their responses to salubrinal and inhibitor XXII were measured. The gBsyn variant contained a single substitution, A855V, in the cytoplasmic tail, and at an MOI of 1, infected cells exhibited 20% fusion at 12 hr post infection (Fig 5A). Fusion levels rose dramatically with increasing concentrations of salubrinal, reaching a maximal 75% (Fig 5A), which is much higher than the response seen with wild-type KOS (Fig 3A). Hence, gBsyn and salubrinal are synergistic. To examine the impact of inhibitor XXII, we used a lower MOI and measured fusion at 24 hr post infection, which enabled gBsyn to reach 37% total fusion in the DMSO control (Fig 5B). As gBsyn prefers to drive fusion with adjacent uninfected cells, the baseline fusion is enhanced at a lower MOI. Treatment with inhibitor XXII dramatically reduced fusion (Fig 5B). These responses of gBsyn emulate what happens to wild-type HSV-1-infected cells when they are exposed to salubrinal and inhibitor XXII.

Syncytial variants of gK cause even higher levels of fusion than those of gB, regardless of the MOI used during infection. Indeed, control cells infected with variants A40V or L118Q exhibited 80% total fusion (Fig 5C and 5D). Because of these intrinsically high levels, we expected salubrinal to have no effect on the gKsyn phenotype; however, that was not the case. Instead, this drug greatly inhibited fusion, particularly in the case of L118Q (Fig 5C). Thus, gKsyn mutants behave similarly to the fusogenic paramyxovirus variant described above. With regard to inhibitor XXII, it did produce the expected result and reduced gKsyn-mediated fusion in a dose dependent manner (Fig 5D). Hence, both gBsyn and gKsyn seem to depend upon PTP1B for their Syn phenotype. Their very different responses to salubrinal is perhaps not surprising since their syncytial phenotypes differ in the requirement for tegument for UL21 [7] and perhaps other accessory proteins.

The Syn variants of UL20 and UL24 have intrinsically weaker phenotypes, but their fusogenic activities were stimulated by salubrinal (Fig 5E and S5 Fig, respectively). However, the maximal amount of fusion (~40%) did not exceed the level observed when the wild-type KOS virus was exposed to salubrinal (Fig 3A). Hence, the alterations that give rise to UL20syn (substitution F222A) and UL24syn (G121A) are not synergistic with salubrinal, and thus, the host factor targeted by this drug seems to work elsewhere on the regulatory machinery of the virus.

Because the UL20syn and UL24syn variants are poorly fusogenic, we did not expect to be able to reliably measure any reductions in syncytia formation resulting from inhibitor XXII. Hence, we were not surprised to find that UL24syn was unaffected by this drug (S5 Fig). However, to our considerable surprise, inhibitor XXII actually stimulated fusion for cells infected with UL20syn (Fig 5F). From this, we predict that the change in UL20 causes a dramatic change to the configuration of the viral machinery such that the substrates of PTP1B are altered.

### PTP1B inhibitor XXII blocks cell-to-cell spread

**Fig 6:**
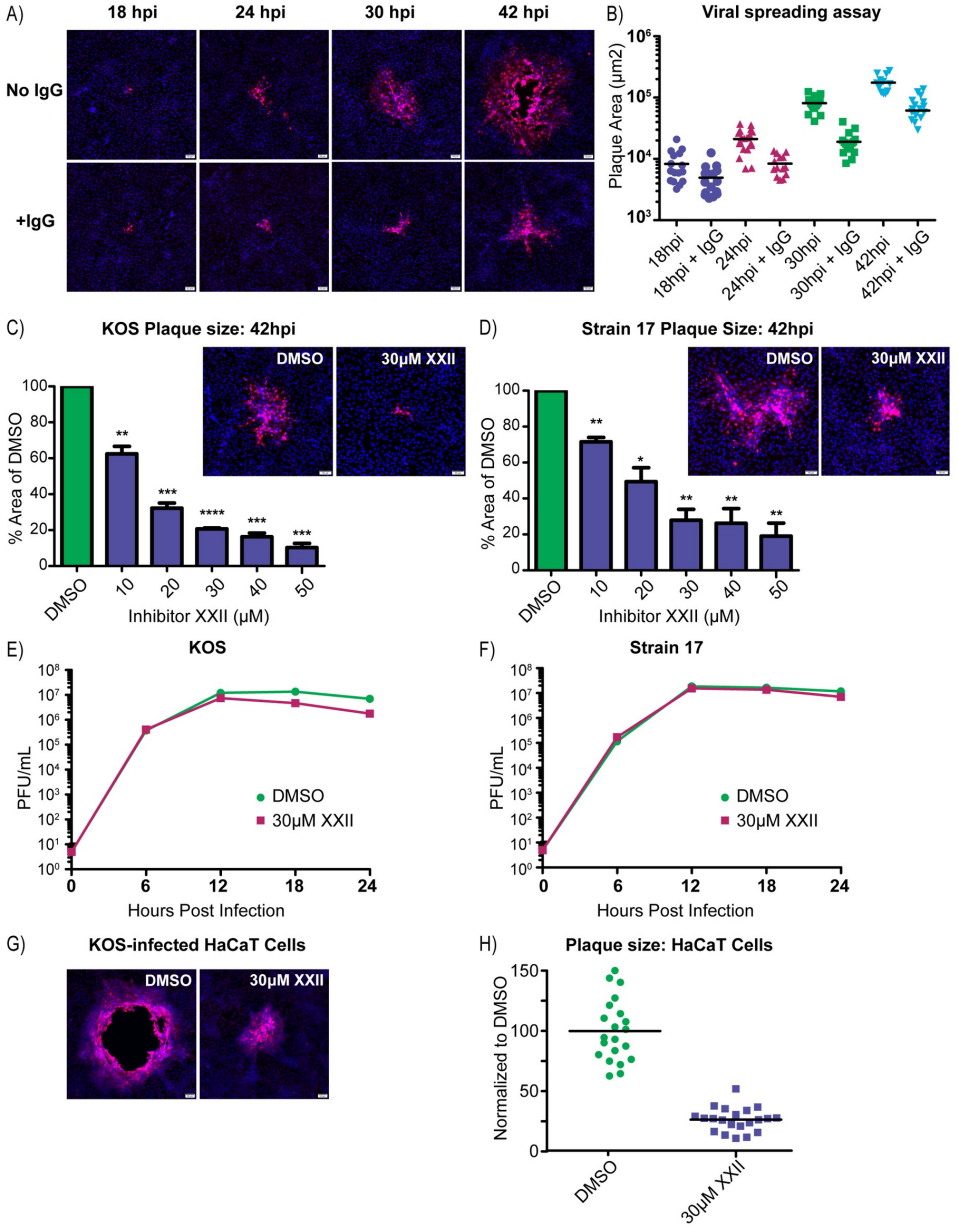
PTP1B inhibitor XXII blocks cell-to-cell spread. **(A)** Vero cells were infected with the KOS strain (100 PFU/well in 6-well plates) and incubated in infection medium or medium containing 5 mg/ml of pooled human IgG to neutralize extracellular virions. At 18, 24, 30, and 42 hpi, the cells were immunostained for the major capsid protein, VP5, and nuclei were stained with DAPI. **(B)** To quantify virus spreading, the areas of 10–15 VP5-positive plaques were measured per sample and these were plotted. **(C and D)** Vero cells were infected (100 PFU/well) with strains (C) KOS or (D) 17 and then treated with increasing amounts of inhibitor XXII, all in the presence of 5 mg/ml of pooled IgG. At 42 hpi, the cells were immunostained for VP5, and representative plaques are shown. The areas of 10–15 plaques were measured for each drug concentration and compared to those from the DMSO control. Data from three independent experiments were combined and are represented as the mean ±SD. A student T-test was used to determine statistical significance for samples compared to the DMSO control. **(E and F)** Virus replication assays were performed in Vero cells infected (MOI = 5) with strains (E) KOS or (F) 17. The cells were incubated in medium containing DMSO or 30 μM inhibitor XXII, and at 6-hour time points, duplicate samples were collected to measure the virus titers (cell lysate + medium), which were averaged and plotted. **(G)** Representative images of plaques produced by the KOS strain on HaCaT cells treated with DMSO or 30 μM inhibitor XXII in the presence of 5 mg/ml pooled IgG. **(H)** At 42 hpi, 20 plaques from each sample in (6G) were measured, and their areas were plotted relative to the average obtained for the DMSO control.

All the experiments up to this point merely show that PTP1B is important for the Syn phenotype, whether induced by salubrinal or viral mutations; however, the overall goal of our experiments was to find host factors that are important for cell-to-cell spread. To examine the importance of PTP1B for this, we used wild-type virus at a low MOI and neutralizing antibodies to prevent cell-free spread. To visualize the sites of infection, the cells were fixed at various times and stained with antibodies against VP5, the major capsid protein. Control experiments without inhibitor XXII (Fig 6A and 6B) confirmed that wild-type virus still forms large plaques via cell-to-cell spread in the presence of neutralizing antibodies, although their development is delayed. In contrast, the plaque sizes for strains KOS and 17 were dramatically decreased as the amount of inhibitor XXII increased (Fig 6C and 6D). We also examined cell-to-cell spread in HaCaT cells, which are a keratinocyte-derived cell line that forms tight junctions and is relevant for HSV-1 infection [40]. With neutralizing antibodies preventing cell-free spread, inhibitor XXII once again dramatically reduced the average plaque size (Fig 6G and 6H).

A reduction in plaque size would also be expected if PTP1B was needed for infectious virion production. To address this possibility, Vero cells were infected at an MOI of 5 and treated with 30 μM inhibitor XXII, which is the minimal dose that gave the maximal effect in the cell-to-cell spread assay (Fig 6C and 6D). The total amount of infectious virus produced was measured over time, and although there was a slight drop in titers at the later time points, the virus titers during the first 12–18 hours were not affected (Fig 6E and 6F). Moreover, the amount of infectious virus released into the culture medium versus that remaining cell associated was unaffected by inhibitor XXII (S6A Fig), and hence, PTP1B is not required for virion egress.

The effect of adding inhibitor XXII prior to attachment and entry of the virus was also examined. In this case, cells were pretreated with DMSO or inhibitor XXII for one hour, and then dilutions of the virus were added for another hour, still in the presence of DMSO or drug. The culture medium was then replaced to remove the drug, and the cells were incubated to allow plaque formation. No reduction in the numbers of plaques was seen with 30 μM inhibitor XXII, and only a small effect was observed at 50 μM (S6B Fig). Collectively, these results suggest that PTP1B activity is not required for binding, entry, replication, or egress. Rather, it appears that inhibitor XXII specifically affects the ability of the virus to move between cells.

### High concentrations of inhibitor XXII can inactivate HSV-1

We further explored the drop in virus titer observed at later time points in the replication experiments. This effect was exaggerated for both strain 17 and KOS when 50 μM of XXII was used, even though the initial rates of infectious virion production were unaffected (S6C Fig). We hypothesized that HSV-1 particles contain discrete binding sites for inhibitor XXII, and as these become saturated, the virus is inactivated. To test this idea, equal amounts of strain 17 were mixed with DMSO or inhibitor XXII, and the virions were incubated at 37°C. Samples were removed at various times and were serially diluted for plaque assays (S6D Fig). Relative to the DMSO control, the 30 μM XXII treatment had no effect on virus titer for the first 12 hours and had only a small effect after that; however, at a concentration of 50 μM, the drug had a dramatic effect, and the virus was completely inactivated by 18 hrs. Although the mechanism of virus killing warrants further investigation, we sought ways to eliminate PTP1B activity without the use of inhibitors.

### Cell-to-cell spread is limited in PTP1B^-/-^ MEFs

**Fig 7:**
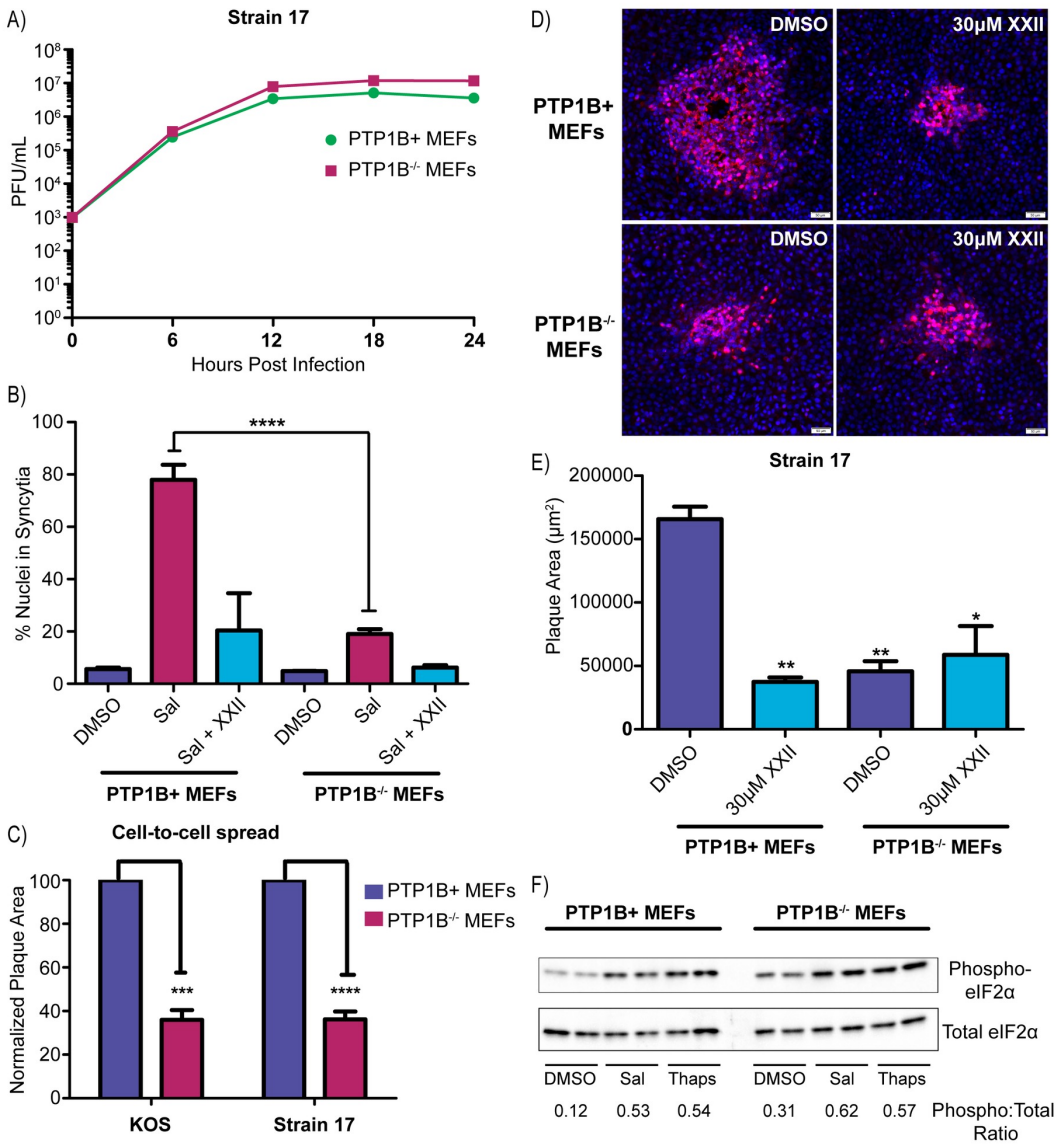
HSV-1 cell-to-cell spread is limited in PTP1B^-/-^ MEFs. **(A)** Virus replication assays were performed in PTP1B+ and PTP1B^-/-^ MEFs infected with strain 17 (MOI = 5). At 6-hour time points, duplicate samples were collected for measurements of the virus titers (cell lysate + medium), which were averaged and plotted. **(B)** To measure syncytia formation, PTP1B+ MEFs or PTP1B^-/-^ MEFs were infected (MOI = 3) with strain 17 and treated with DMSO, 50 μM salubrinal, or 50 μM salubrinal + 30 μM inhibitor XXII. At 24 hpi, cells were immunostained for ZO-1 and DAPI-stained nuclei in syncytia were manually counted. 1000 nuclei were scored per image, and 2 replicates were averaged. **(C)** To assay for cell-to-cell spread, PTP1B+ MEFs or PTP1B^-/-^ MEFs were infected (100 PFU/well) with strains KOS or 17, and the cells were incubated in medium containing 5 mg/ml pooled IgG. At 42 hpi, the cells were immunostained for VP5, and the areas of 10–15 plaques per sample were measured. Data are represented as mean ±SD from 3 independent experiments. A student T-test was used to determine statistical significance for the PTP1B^-/-^ samples compared to the PTP1B+ control. **(D and E)** PTP1B+ MEFs or PTP1B^-/-^ MEFs were infected with strain 17 (100 PFU/well) and incubated in medium containing 5 mg/ml of pooled human IgG along with either DMSO or 30 μM inhibitor XXII. At 42 hpi, cells were immunostained for VP5. (D) Representative plaques are shown. (E) To quantify the results, 10–15 plaques per sample were measured, and the mean plaque area was plotted from 2 independent experiments. A student T-test was used to determine statistical significance for samples compared to the PTP1B+ DMSO control. **(F)** To ascertain their ability to respond to salubrinal, uninfected PTP1B+ MEFs or PTP1B^-/-^ MEFs were incubated in medium containing DMSO, 50 μM salubrinal, or 1 μM thapsigargin for 2 hours. Cell lysates were harvested in the presence of phosphatase inhibitors and probed via western blotting for total eIF2α and phosphorylated eIF2α. Duplicate samples were analyzed in the same western blot and band intensities were quantified.

Fortunately for this investigation, PTP1B-knockout mice have been made and found to develop into adults [41, 42]. Moreover, immortalized mouse embryo fibroblasts (MEFs) have been derived from these mice (PTP1B^-/-^), along with a reconstituted line (PTP1B+) in which the coding sequence has been reinserted with a retroviral vector [43]. These two lines were obtained, and immunoblots with an antibody specific for PTP1B confirmed their identities (S7 Fig). If PTP1B is not required for HSV replication, then there should be no defect in viral replication on PTP1B^-/-^ cells, and that is what we found (Fig 7A). Also, PTP1B+ cells infected with strain 17 exhibited robust cell fusion when treated with salubrinal, although the development of syncytia took a few hours longer than with Vero cells, and this was blocked by inhibitor XXII (Fig 7B). As expected, the knockout cells were not very responsive to salubrinal, and although a small induction of fusion was observed, this was eliminated with inhibitor XXII (Fig 7B). Hence, another tyrosine phosphatase similar to PTP1B (e.g., TC-PTP) [44] might be able to compensate.

To test the ability of the two MEF lines to support cell-to-cell spread, they were infected at low MOI with strain 17 or KOS, and neutralizing antibodies were added to the medium to block cell-free spread. At 42 hr post infection, the average plaque size on PTP1B^-/-^ cells was reduced by more than 60% for both viruses compared to their sizes on reconstituted PTP1B+ cells (Fig 7C). A somewhat greater reduction in plaque size was seen when strain 17-infected PTP1B+ cells were treated with inhibitor XXII, but there was no further decrease in plaque size in the parallel culture of PTP1B^-/-^ cells (Fig 7D and 7E). These experiments, along with the inhibitor XXII studies in Vero and HaCaT cells, confirm that PTP1B is required for cell-to-cell spread of HSV-1. To try and gain mechanistic insight, KOS-infected HaCaT cells were treated with DMSO or XXII and gE, E-Cadherin, and VP5 were examined with confocal microscopy. We found that gE (S8A Fig) and E-Cadherin (S8B Fig) localization was unaffected by PTP1B inhibition, and gE trafficking to the cell junctions (where it is known to accumulate) was not impeded. Additionally, VP5 accumulation at cellular junctions did not appear to be increased in cells treated with inhibitor XXII (S8B Fig). Ultimately, it appears that PTP1B inhibition does not block cell-to-cell spread by mislocalizing gE or altering capsid trafficking.

### PTP1B is not the target of salubrinal

The small increase in cell fusion observed when infected knockout cells were treated with salubrinal (Fig 7B) suggested that the cellular target of this drug might still be present. If PTP1B is not the target, then eIF2α should be phosphorylated to the same extent in response to salubrinal in both MEF lines. For this experiment uninfected cells were used, and these were treated with DMSO, salubrinal, or thapsigargin, which is a well-known inducer of ER stress [45]. The basal level of eIF2α phosphorylation was higher for the knockout cell line (Fig 7F), but this was expected since PTP1B is needed to fully shut down the eIF2α kinase PERK [46]. Thapsigargin-induced stress pushed both cell lines into states with equally high levels of phosphorylation, and these levels were matched by salubrinal treatment (Fig 7F). Thus, PTP1B seems unlikely to be the target of salubrinal.

## Discussion

The results described here are broadly significant for several reasons. They show that mechanistic studies of HSV-1 syncytia formation can provide important clues for elucidating the more relevant biological process of cell-to-cell spread. In particular, we have shown for the first time that tyrosine phosphorylation regulates the machinery of cell-to-cell spread (and syncytia formation), and at least two distinct host factors are involved; one being phosphatase PTP1B, which could not have been deduced from the previous literature. This finding may have immediate utility since an inhibitor of PTP1B (MSI-1436) has passed early clinical trials for treatments of type-2 diabetes, obesity, and cancer [47], and perhaps this drug can be used to attenuate HSV-1 reactivation disease in immunocompromised patients. The discoveries presented here also illuminate several paths forward for studies of the spreading mechanism of HSV-1. All these points are discussed below in context of the mechanism of cell-to-cell spread.

### Prevailing model for cell-to-cell spread

HSV-1 capsids are wrapped with host-derived membranes in the cytoplasm to produce mature virions within vesicles [48], and these subsequently fuse with the plasma membrane to release their contents for cell-free spread. As shown here, PTP1B is not required for any of the steps in that pathway, but instead is involved only in cell-to-cell spread. The prevailing model for cell-to-cell spread merely requires transport of virion-containing vesicles to lateral cell junctions, where fusion with the plasma membrane releases HSV-1 into intercellular spaces so that the adjacent cells can be infected [20]. This simple model does not specify how many virions can be delivered into the adjacent cells or whether those cells have a mechanism for excluding the passage of subsequent virions, as is the case for cell-free infections, where the “door closes” two hours after the initial infection [49]. Also, this model does not specify any role for viral proteins on the plasma membrane within cell junctions.

In support of the current model, mature virions are observed in spaces between cells [50]. Also, receptor nectin-1 resides in cell junctions, where the virus can bind (via gD) to gain entry into adjacent cells [51]. However, because HSV-1 downregulates nectin-1 in infected cells, this receptor is only present on the adjoining uninfected cell [52]. Consequently, virions released into lateral junctions (as opposed to viral proteins on the plasma membrane) are unlikely to be the drivers of fusion for Syn mutants or in salubrinal treatments because they cannot fuse with the cell from which they emerged [52]. Further supporting the current cell-to-cell spread model, the tail of gE, which forms a heterodimer with gI (Fig 1A) and is likely exposed on the cytoplasmic faces of virion-containing vesicles [53], appears to be required for transport to lateral junctions [54]. That is, mutants lacking the tail of gE have been reported to release their virions at apical membranes rather than at cell junctions [50, 54].

There are many reasons to suspect that cell-to-cell spread is more complicated than merely transporting virions to cell junctions. For example, the list of viral proteins that seem to participate in the spreading mechanism is quite long and includes (just for example) four tegument proteins that directly interact with the tail of gE, namely UL11, UL16, VP22, and UL51 [18, 19, 55]. The current model for cell-to-cell spread does not take into account this complexity. Unfortunately, mutational studies of these and all the other viral proteins that have been implicated in the spreading mechanism (including gE/gI) are difficult to interpret because these proteins play multiple roles in the virus replication cycle (capsid envelopment, for one example). Moreover, these proteins exist in poorly-defined complexes with one another, and those can fall apart when all or a portion of one subunit is missing [56, 57]. Also, the viral proteins that mediate cell-to-cell spread may assemble into unique complexes with unexpected properties. For instance, pseudorabies virus (PRV, another neurotropic herpesvirus) does not require gD for cell-to-cell spread, even though this glycoprotein is essential for infectivity. That is, a null mutant propagated in complementing cells to temporarily provide gD, can infect cell cultures or pigs, where it spreads cell-to-cell efficiently, but all of the progeny viruses lack gD and are noninfectious for cell-free spread [58, 59]. Thus, at least for PRV, the machinery used for cell-to-cell spread is not identical to that used for virus entry.

### Roles for viral proteins on the plasma membrane in cell junctions

There is strong evidence that the membranes used to wrap capsids in the cytoplasm are derived from endosomes, and therefore, it is likely that these carry all the viral membrane proteins, which would have been at least transiently present on the cell surface [60]. Moreover, studies too numerous to cite have shown that many viral proteins indeed accumulate on the plasma membrane within cell junctions. For example, detailed studies have shown that gE/gI and gB are redistributed to cell junctions at late times after infection [61], and here we have shown that gE is present, even when PTP1B is inactive. Of particular relevance (explained below), gN/gM complexes are also found at cell junctions [62]. In addition to directing its own proteins to cell junctions, HSV-1 induces a remodeling of cellular proteins in adherens junctions. For example, the connections provided by nectin-1 subunits, which dimerize to link cells, are lost when gD downregulates the subunits in infected cells, thereby freeing up their partners in adjacent cells to allow infection [51, 52, 63]. Also, cellular membrane proteins TGN46 and carboxypeptidase D have been reported to accumulate at cell junctions [61].

A role for modified-cell junctions has been long imagined; in particular with regard to gE/gI [20, 64], but it remains unclear what is accomplished for the mechanism of cell-to-cell spread. It is not obvious why viral fusion machinery would be needed at cell junctions to support the release of virions contained within secretory vesicles. Outside of the herpesvirus family, measles virus induces the formation of an intercellular pore, which facilitates the transfer of cytoplasm from infected to uninfected cells and potentially provides a pathway for the direct transfer of virions between cells [2, 65]. While there is no evidence that HSV capsids pass through specialized pores during cell-to-cell spread, it cannot be ruled out, and it is intriguing that neurons infected with PRV form small pores between cells in a manner that is dependent upon the viral fusogen, gB [66].

Viral membrane proteins may also be needed to create contacts between cells where they do not normally exist. This is perhaps easiest to understand with sensory neurons, which HSV-1 enters to establish latent infections and leaves after reactivation, employing cell-to-cell spread in both directions. However, sensory neurons respond to mechanical forces and temperature (for example) but do not normally establish synapses with epithelial cells [67]. Thus, it seems likely that viral proteins on the surface of infected cells are needed to establish connections to enable the passage of HSV-1, perhaps in a manner analogous to the unique connections created by HIV for cell-to-cell spread [68]. In this regard, it is interesting that PTP1B plays a role in cell adhesion and migration [69, 70], which may permit infected cells to make local changes in position to establish contacts with nearby uninfected cells. However, cell-to-cell spread occurs in cells that already do have cell junctions, and it is likely that unique, virus-specific connections are needed in this situation, too.

Of course, the presence of viral fusion machinery at cell junctions requires that it be tightly regulated; otherwise, syncytia would form. While the regulatory mechanism is virtually unknown, our experiments confirm that salubrinal treatments disrupt it, but they go further in showing that tyrosine phosphorylation plays a critical role. In addition, the differential responses of the four types of Syn mutants to salubrinal and PTP1B inhibitor XXII (illustrated in S9 Fig) make it clear that they each dysregulate the viral fusion machinery in distinct ways, all of which deserve further investigation.

### Mechanistic role for PTP1B in cell-to-cell spread

PTP1B has been shown to be anchored in the endoplasmic reticulum with its catalytic domain positioned in the cytosol where it modulates a wide variety of cellular functions, including the unfolded protein response [36, 71, 72]. We cannot rule out the possibility that PTP1B plays a role in the transport, docking, or fusion of vesicles at cell junctions so that their virion cargoes can be released. We did not observe an obvious accumulation of capsids at cell junctions by confocal microscopy in the absence of PTP1B activity, but that might be expected if transport to cell junctions was impaired. However, the importance of this enzyme in influencing the properties of Syn mutants and salubrinal treatments strongly suggests that it plays a role in modulating viral machinery on the plasma membrane within cell junctions. This is not surprising because PTP1B is well known to regulate cellular receptors on the plasma membrane [73] and is recruited to regions of cell-cell contact to bind N-cadherin and stabilize its association with beta-catenin at adherens junctions [74, 75].

In view of our findings, it is intriguing that gM, which also regulates the viral fusion machinery at cell junctions along with its disulfide-bonded partner, gN [62], has very recently been found to interact with “extended synaptotagmin 1” [76]. This host protein helps connect the ER to the plasma membrane [77], and it might play a role in positioning PTP1B at the proper sites for its regulatory activity in the cell-to-cell spreading mechanism.

### Future studies of the role of PTP1B in cell-to-cell spread

There are three critical areas that need to be pursued to further elucidate how PTP1B participates in the spreading mechanism. One is the identification of the proteins that contain the critical tyrosine substrates. Fortunately a PTP1B trapping mutant is available that can bind but not dephosphorylate [78]. Insertion of the coding sequence for this mutant into the wild-type HSV-1 genome would allow it to be expressed in all infected cells, and if a suitable tag is fused to the construct, then the bound substrates can be recovered and identified by mass spectrometry with known cellular targets of PTP1B serving to validate the approach [79]. Viral substrates would be particularly interesting, as would any novel cellular substrates, all of which would have to be confirmed as being important for cell-to-cell spread and syncytia formation.

Another critical question is whether the absence of PTP1B blocks cell-to-cell spread of wild-type HSV-1 in animals. Mouse lines are already available in which the gene for PTP1B is knocked out in all tissues [41], and other lines are available that have the gene flanked by LoxP sites so that expression can be knocked out in specific tissues [80], with neuronal and epithelial cells being of particular interest. There are well-established methods for investigating HSV infection *in vivo* [81, 82], and it should be straightforward to determine whether and how the absence of PTP1B affects cell-to-cell spread *in vivo*. It is possible that this enzyme would only be needed in infected cells, but perhaps it is needed in target cells, too. If PTP1B is in fact needed for cell-to-cell spread in mice, then existing inhibitors (e.g., MSI-1436, see above) might prove to be beneficial for treatment of HSV-1 reactivation disease in immunocompromised patients.

One of the biggest missing pieces to this story is the target of salubrinal. Our initial candidate was PTP1B, but when this host protein is absent, phosphorylation of eIF2α still occurs normally in response to salubrinal. Our data suggest that the target for salubrinal is contained within a protein complex that includes PTP1B, and it is possible that it will turn out to be a tyrosine kinase. However, if the target of salubrinal is not a kinase, then there must be three host factors involved: PTP1B, a tyrosine kinase, and the target of salubrinal.

Lastly, all herpesviruses have mechanisms for cell-to-cell spread, none of which are understood. It is possible that the host factors implicated here in the spreading mechanism of HSV-1 may be relevant for other viruses. Indeed, our data suggest that PTP1B and the target of salubrinal may even play a role in paramyxoviruses.

## Materials and methods

### Cells and viruses

Vero (African green monkey kidney) cells (a gift from Richard Courtney, Penn State University) were grown in Dulbecco’s modified Eagle’s medium (DMEM; Gibco) supplemented with 5% bovine calf serum (BCS; HyClone), 5% fetal bovine serum (FBS; HyClone), and penicillin-streptomycin (pen/strep; Gibco). HaCaT (human) cells (a gift from Craig Meyers, Penn State University), C10 (mouse) cells (a gift from Gary Cohen, University of Pennsylvania), and BHK-21 (Syrian golden hamster) cells (a gift from Nicholas Buckovich, Penn State University) were grown in DMEM supplemented with 10% FBS and pen/strep. MDA-MB-231 (human) breast cancer cells (ATCC, Manassas, VA) were grown in DMEM with 10% FBS and pen/strep. PTP1B^-/-^ mouse embryonic fibroblasts (MEFs) (a gift from Benjamin Neel, NYU) were cultured in DMEM supplemented with 10% FBS and pen/strep, and the recombinant PTP1B^+^ MEFs also received hygromycin B (200 μg/mL; ThermoFisher) to select for the retroviral vector [43]. All cells were maintained in a humidified incubator at 37°C in 5% CO_2_. Infected cells were cultured in DMEM supplemented with 1% FBS.

The KOS and F strains of HSV-1 were produced by transfecting Vero cells with BACs containing the viral genomes [83] and harvesting the virus produced by transfected cells (transfection stock). Virus stocks were generated by infecting Vero cells with the transfection stocks at an MOI of 0.01 and harvesting at 48 hours post by freezing and thawing the cultures 3 times to maximize the release of virions. A virus stock of strain 17 was a gift from Moriah Szpara (Pennsylvania State University). HSV-1 mutants ΔUL16, gEΔCT, gB.A855V, gK.A40V, gK.L118Q, UL20.F222A, and UL24.G121A were previously constructed [7, 18, 29] in the KOS genome by means of BAC recombineering [84]. A virus stock of the PRV Becker strain was a gift from Lynn Enquist (Princeton University). Titers of all HSV variants and PRV were measured by plaque assays on Vero cells. Virus stocks of wild-type PIV5 and mutant rPIV5-NPΔ4 were produced as previously described [39].

### Cell fusion assays

For experiments with HSV-1 and PRV, Vero cells or BHK-21 cells were seeded into 6-well plates at a density of 4.2 x 10^5^ cells/well and cultured for 2 days. Once confluent, the cells were infected at the indicated MOIs, which ranged from 0.1 to 3 depending on the experiment. During the 1-hour infection period, cells were incubated at 37°C and rocked every 15 minutes. After infection, the cells were rinse once with DMEM and incubated at 37°C with media containing DMSO (5 μl/ml) (Sigma-Aldrich), salubrinal (Sigma-Aldrich), or inhibitor XXII (CAS 765317-72-4; Santa Cruz). Experiments were conducted with equal volumes of DMSO in each sample, reaching a final concentration of 5 μl/ml of DMSO. Incubation time ranged from 12–24 hours depending on the experiment. Because fusion was slower to develop in PTP1B^-/-^ MEFs, measurements were taken at 24 hours post infection.

In the paramyxovirus experiments, Vero cells were plated at 1.2x10^6^ cells per well in 6-well dish one day prior to infection with rPIV5 NPΔ4v6 at MOI of 0.1 or rPIV5 at MOI of 1. Viruses were allowed to adsorb for one hour at 37°C, and then the cells were washed twice with 1X PBS, followed by replacement with high-glucose DMEM supplemented with 2% FBS, pen/strep, and either DMSO, 30 μM PTP1B inhibitor XXII, or 50 μM salubrinal. Cells were returned to the incubator and syncytia formation monitored. At various times post infection, monolayers were visualized using a Nikon Eclipse TS100-F microscope and photographed using a Nikon DS-Fi1 digital camera.

### Quantification of cell fusion

To manually quantify the amount of cell fusion, cells were fixed for 10 minutes in 4% paraformaldehyde (PFA; Sigma-Aldrich), rinsed two times in PBS, permeabilized with 0.1% Triton X-100 for 10 minutes, and subsequently blocked for 30 minutes in 2% BSA/PBS (Sigma-Aldrich). To visualize the cell perimeters, the samples were incubated with a mouse monoclonal antibody specific for ZO-1 (1A12; ThermoFisher) at a dilution of 1:500 for one hour. After rinsing 2X with PBS, the samples were stained with a fluorescently-conjugated secondary antibody (g-α-m Alexa568; Life Technologies) at a dilution of 1:1000 for one hour, and nuclei were stained with DAPI (Molecular Probes) for 5 minutes. Images were captured with an Olympus IX73 inverted microscope. Fusion was calculated by manually counting the number of nuclei present in single cells versus the number of nuclei contained within syncytia with the aid of Olympus cellSens software. At least 1000 nuclei were scored per image for each experimental replicate.

To quantify cell fusion in a more rapid and unbiased manner, a novel flow cytometry method was developed. For this, cultures in 6-well plates containing syncytia were gently rinsed with standard buffer, and then 400 μl of trypsin in standard buffer (Sigma-Aldrich) was added to each well. The plate was rocked every minute until the majority of cells were lifting off the plate, and then the cells were fixed by adding 600 μl of ice-cold 4% PFA/PBS solution (EMS; Corning) to each well for a total volume of 1ml. Importantly, all buffers used were Ca^2+^/Mg^2+^-free to minimize cell clumping. The solution was pipetted up and down around the entire well 10–20 times to break up syncytia and achieve a homogenous suspension. The samples were transferred to pre-chilled flow cytometry tubes on ice, and the wells were rinsed with 200 μl of FACS buffer (2% BSA, 3 mM EDTA in Ca^2+^/Mg^2+^-free PBS), which was added to the respective sample tubes. Nuclei were stained by adding 200 μl of propidium iodide staining solution (100 μg/ml in FACS buffer; ThermoFisher) to each sample. Flow cytometry tubes were vortexed for 3 seconds, placed on ice, and covered with foil to limit light exposure. The samples were passed through a BD LSRFortessa cell analyzer within 1 hour of harvesting. 50,000 PI-positive events were collected per sample, and the data were analyzed with FlowJo software. Briefly, PI-positive events were gated for FSA (forward scatter) and by SSC (side scatter) using a logarithmic scale. Events were then categorized as free nuclei or intact cells having single nuclei based on size and granularity (S1 Fig). The percentage of free nuclei was used to calculate total cell fusion within a sample.

### Virus replication assays

Vero cells were seeded in 6-well plates at a density of 4.2 x 10^5^ cells/well, and after reaching confluency (~1.2 x 10^6^ cells/well), infections were initiated with KOS or strain 17 at an MOI of 5 in a volume of 500 μl for 1 hour at 37°C. To inactivate virions remaining on the cell surface, the cultures were briefly rinsed with a low pH buffer (135mM NaCl, 10mM KCl, 40mM citric acid, pH 3.0) and then rinsed two times with DMEM. The cells were then incubated at 37°C in 1 ml/well of DMEM (+1% FBS) containing DMSO or the specified amounts of salubrinal, guanabenz (Sigma-Aldrich), or inhibitor XXII. At 6, 12, 18, 24, and 30 hours post infection, a cell scraper was used to harvest the total cells and media for each sample. Not all time points were used for every experiment and differences are noted in the accompanying figure legends. Each sample was processed through 3 freeze/thaw cycles prior to serial dilution and titration by plaque assay. For MEFs, infections were started when cell density reached 1.3 x 10^6^ cells/well and a citric acid wash was not used. For the virus egress assay, infected cells and media were harvested and titered separately.

### Cell-to-cell spreading assay

Vero cells, HaCaT cells, or MEFs were seeded onto glass coverslips in 6-well plates, allowed to reach confluency, and infected with ~100 PFU/well of KOS or strain 17. After 1 hour, the cells were rinsed twice with DMEM and incubated in infection media (DMEM + 1% FBS) containing 5 mg/ml pooled human IgG (Equitech-Bio, Inc). This concentration of IgG was previously determined to neutralize all but 2 virions per 1x10^6^ PFU [7]. At 42 hpi, the cells were fixed for 10 minutes in 4% paraformaldehyde (PFA; Sigma-Aldrich), rinsed twice in PBS, permeabilized with 0.1% Triton X-100 for 10 minutes, and blocked for 30 minutes in 2% BSA/PBS (Sigma-Aldrich). The samples were stained with a rabbit antibody against VP5 (the major capsid protein) at a dilution of 1:1000 for 1 hour, rinsed 3 times with PBS, and stained with an Alexa 568 fluorescent secondary antibody (Life Technologies) for 1 hour at a dilution of 1:1000. Finally, samples were stained with DAPI for 5 minutes, rinsed 3X with PBS, and the coverslips were mounted on slides using Aqua Poly/Mount (Polysciences, Inc.). Fluorescent images of plaques were captured using an Olympus IX73 inverted microscope, and their sizes were measured using Olympus cellSens software.

### Identification of PTP1B

A biotinylated derivative of salubrinal, UTX-102 (S2A Fig), was synthesized and is similar to ones previously described [85]. MDA-MB-231 cells were incubated with 20 μM UTX-102 for 5 hours, and cell pellets were lysed in buffer (100 mM Tris-HCl pH 7.5, 150 mM NaCl, 1% Triton X-100, and 5% glycerol) supplemented with phosphatase (Sigma-Aldrich, St. Louis, MO, USA) and protease (Thermo-Fisher Scientific, Waltham, MA, USA) inhibitors. Avidin-biotin pull-downs were carried out with a Pierce Monomeric Avidin kit (Thermo-Fisher Scientific, Waltham, MA, USA). Briefly, samples were incubated in the column to which the monomeric avidin was immobilized, and these were washed with PBS until absorbance at A280 came to baseline, indicating that most non-specific binding proteins were removed. Bound proteins were eluted, concentrated, desalted, denatured, and alkylated according to standard protocols. The samples were digested with trypsin overnight (37°C), and peptides were desalted with a Silica C18 MacroSpin Column, reconstituted in 5% ACN, 0.1% formic acid, and injected onto a ODS-100V C18 column (Tosoh Bioscience). The peptides were eluted with a linear gradient, and the effluent was electro-sprayed into a LTQ Orbitrap mass spectrometer. Blanks were run prior to the sample run to ensure that no significant background signals existed from solvents or the columns. Database search and data analysis were performed via Thermo-Fisher Scientific Proteome Discoverer^™^ software (v1.3) against Uniprot protein database (Uniprot_072413_HUMAN.fasta).

### Immunoblotting

To detect PTP1B, cells were lysed in standard RIPA buffer containing protease inhibitors. Proteins were separated in a SDS-10% polyacrylamide gel and electro-transferred to Immobilon-P or nitrocellulose membranes, which were incubated first with a primary antibody against PTP1B (rabbit polyclonal, #5311S; Cell Signaling) and then with a secondary antibody conjugated with horseradish peroxidase. Protein levels were assayed with a SuperSignal West Femto Maximum Sensitivity substrate (Thermo Scientific) or Pierce West Pico PLUS reagents.

To assess the phosphorylation state of eIF2α, Vero cells or MEFs were treated with DMSO, salubrinal, guanabenz, or thapsigargin as indicated, and the cells were lysed on ice for 10 minutes in protein extraction buffer (10 mM Tris hydroxymethyl aminomethane, pH 6.8, 150 mM NaCl, 1 mM EDTA, and 0.5% Igelpal; Sigma) containing protease (Sigma #P8340) and phosphatase (PhosStop; Roche) inhibitor cocktails at recommended concentrations. Debris was removed with a 21K x g spin at 4°C for 10 minutes, and the protein concentration of each supernatant was measured by the Pierce BCA assay. Samples containing 20 μg were electrophoresed in an SDS-12% polyacrylamide gel, and transferred to a sheet of nitrocellulose, which was subsequently blocked with 5% BSA in TBS-T (Tris-buffered saline, pH 7.6, 0.1% Tween 20). The total amounts of eIF2α were measured with a 1:1000 dilution of a primary mouse monoclonal antibody (#2103S; Cell Signaling Technology) and a secondary HRP-conjugated goat-anti-mouse antibody. The levels of phosphorylated eIF2α were measured with a 1:1000 dilution of a primary rabbit monoclonal antibody (#3398S; Cell Signaling Technology) and a secondary HRP-conjugated goat-anti-rabbit antibody. Chemiluminescence signals were detected with Pierce West Pico PLUS reagents using BioRad ChemiDocMP and quantified with ImageLab 6.0 Software.

### Visualization of proteins at cell junctions

HaCaT cells were seeded onto coverslips and infected with the KOS strain at an MOI of 0.1. Cells were incubated in infection media containing DMSO or 30 μM drug XXII for 18 hours at 37°C, fixed in 4% paraformaldehyde (PFA; Sigma-Aldrich) for 10 minutes, and rinsed twice in PBS. Then, they were permeabilized with 0.1% Triton X-100 for 15 minutes, blocked for 1 hour in 2% BSA/PBS (Sigma-Aldrich), and exposed for 1 hour to primary antibodies against gE (mouse monoclonal 3114, kindly provided by David Johnson, Oregon Health & Science University) at a 1:4000 dilution, E-cadherin (mouse monoclonal, #610818; BD Biosciences) at a 1:300 dilution, or VP5 (rabbit polyclonal) at a 1:1000 dilution. After rinsing 3 times with PBS, the cells were stained with secondary fluorescent goat antibodies (Alexa 488-anti-mouse or Alexa 568-anti-rabbit; Life Technologies) and DAPI (Molecular Probes). The coverslips were mounted onto slides with Aqua Poly/Mount (Polysciences, Inc.), and Z-stack images were obtained with a Nikon C2+ confocal microscope and processed using Nikon Elements software.

### Quantification and statistical analysis

All statistical analyses were performed using Prism (GraphPad, v4). A two-tailed Student’s T-test was used to determine statistical significance and the number of replicates performed for each experiment is listed in the figure legends. FlowJo was used to analyze all flow cytometry data, Nikon Elements was used to process confocal images, and Olympus cellSens was used for manual counting of nuclei.

## Supporting information

S1 Fig Flow cytometry analysis of fused cells. Samples from strain 17-infected cells were prepared and run through a BD LSRFortessa cell analyzer as described in (Fig 2A and 2B). These dot plots represent the PI-positive events as measured by SSC-A and FSC-A. The intact cell population and free nuclei population are denoted by the drawn circles and accompanying percentages.
(TIF)

S2 Fig PTP1B pulls down with a biotinylated analogue of salubrinal. **(A)** Diagram of salubrinal analogue, UTX-102. Segments representing salubrinal and biotin are indicated. **(B)** MDA-MB-231 cells were treated with DMSO, salubrinal, or UTX-102 in increasing amounts. Cell viability was measured and the cell survival ratio calculated. **(C)** MDA-MB-231 cells were treated with 20 μM UTX-102 for 5 hours, and total cell proteins were harvested in the presence of phosphatase and protease inhibitors. An avidin-biotin pull-down assay was utilized, and PTP1B was shown to be present in the complex by western blot analysis. Input is listed as PC (protein control) and a biotin-only control was also utilized.
(TIF)

S3 Fig Core fusion machinery transfections. C10 cells were transfected with the core fusion plasmids (gB, gD. gH/gL) in a 3:1:1:1 ratio. Transfected cells were treated with DMSO, 50 μM salubrinal, or 50 μM inhibitor XXII. These images were taken at 24 hours post transfection.
(TIF)

S4 Fig Effects of salubrinal and inhibitor XXII on a paramyxovirus. **(A and C)** Vero cells were infected with wild-type PIV5 at an MOI of 1 and incubated in medium containing DMSO, 50 μM salubrinal, or 30 μM inhibitor XXII, as indicated. Images were taken 96 hpi. Examples of syncytia are indicated with arrows. **(B and D)** Vero cells were infected with fusogenic mutant rPIV5-NPΔ4v6 at an MOI of 0.1 and incubated with DMSO, 50 μM salubrinal, or 30 μM inhibitor XXII, as indicated. Images were taken at 24 hpi.
(TIF)

S5 Fig Effects of salubrinal and inhibitor XXII on UL24syn-infected cells. Vero cells were infected with Syn mutant UL24.G121A at an MOI of 3 and incubated with DMSO, 50 μM salubrinal, or 50 μM inhibitor XXII. At 12 hpi, the cells were harvested and fusion was assessed by flow cytometry. The averages from two independent experiments are shown.
(TIF)

S6 Fig Effects of inhibitor XXII on virus egress, entry, and infectivity. **(A)** Vero cells were infected with strain 17 (MOI = 5) and treated with DMSO or 30 μM inhibitor XXII. At 6 and 12 hpi, infected cell lysates and media were harvested separately, and the virus titers were measured for each. **(B)** Vero cells were treated with DMSO, 30 μM inhibitor XXII, or 50 μM inhibitor XXII. After 1 hour of treatment, cells were infected for another hour with serial dilutions of strain 17 in medium also containing DMSO or inhibitor XXII. The cells were then rinsed 2 times and overlaid with methylcellulose for 3 days, and the titers were calculated and represented as mean ±SD from 3 independent experiments. **(C)** Virus replication assays were performed in Vero cells infected (MOI = 5) with strains KOS or 17, which were incubated in medium containing DMSO or 50 μM inhibitor XXII. At 6-hour time points, duplicate samples were collected to measure the virus titers (cell lysate + medium), which were averaged and plotted. **(D)** Three identical tubes containing 1x10^7^ pfu/ml of strain 17 received either DMSO, 30 μM inhibitor XXII, or 50 μM inhibitor XXII. The tubes were incubated at 37°C, and duplicate samples were collected at the indicated times. The amount of infectious virus present in each sample was measured by plaque assay, and the duplicate measurements were averaged.
(TIF)

S7 Fig Expression of PTP1B in MEFs. To verify the MEF cell lines used in this study, lysates of PTP1B^-/-^ and PTP1B^+^ cells were prepared and analyzed by western blotting with PTP1B-specific antiserum. GAPDH was used as a loading control.
(TIF)

S8 Fig Localization of gE and E-cadherin are unaffected by inhibitor XXII. **(A)** HaCaT cells were infected (MOI = 0.1) with the KOS strain and incubated in the presence of DMSO or 30 μM inhibitor XXII. At 18 hpi, the cells were fixed and immunostained for gE while nuclei were stained with DAPI. Images were taken with a Nikon C2+ confocal microscope, and Z-stacks were collected. Images of representative slices are shown (scale bars indicate 25 μm). **(B)** HaCaT cells were infected and treated with DMSO or inhibitor XXII as described in (S8A) and were immunostained for E-cadherin or VP5 while nuclei were stained with DAPI. The images for E-cadherin are from one slice of the Z-stack while the VP5 images show the maximum projection of the same Z-stack.
(TIF)

S9 Fig Summary of the responses to salubrinal and PTP1B inhibitor. For cells infected with wild-type HSV-1 (top panels), salubrinal stimulates fusion, but this is blocked by the PTP1B inhibitor. By itself, the PTP1B inhibitor blocks cell-to-cell spread. For the four different types of syncytial viruses (remaining panels), the effects of the two drugs depend on which Syn mutant is used. Unexpectedly, salubrinal blocks cell fusion for gKsyn mutants, and inhibition of PTP1B stimulates fusion of cells infected with UL20syn mutants. Collectively, these findings reveal for the first time that tyrosine phosphorylation and dephosphorylation are critical regulators of the viral machinery involved in cell-to-cell spread. Syn mutants dysregulate the machinery and are also critically dependent upon tyrosine phosphorylation and dephosphorylation.
(TIF)
