# Supplementary figures and images for: The HSV-1 mechanisms of cell-to-cell spread and fusion are critically dependent on host PTP1B
Source: PLoS Pathog. 2018 May 9;14(5):e1007054. doi: 10.1371/journal.ppat.1007054 (PMC5962101; doi:10.1371/journal.ppat.1007054)

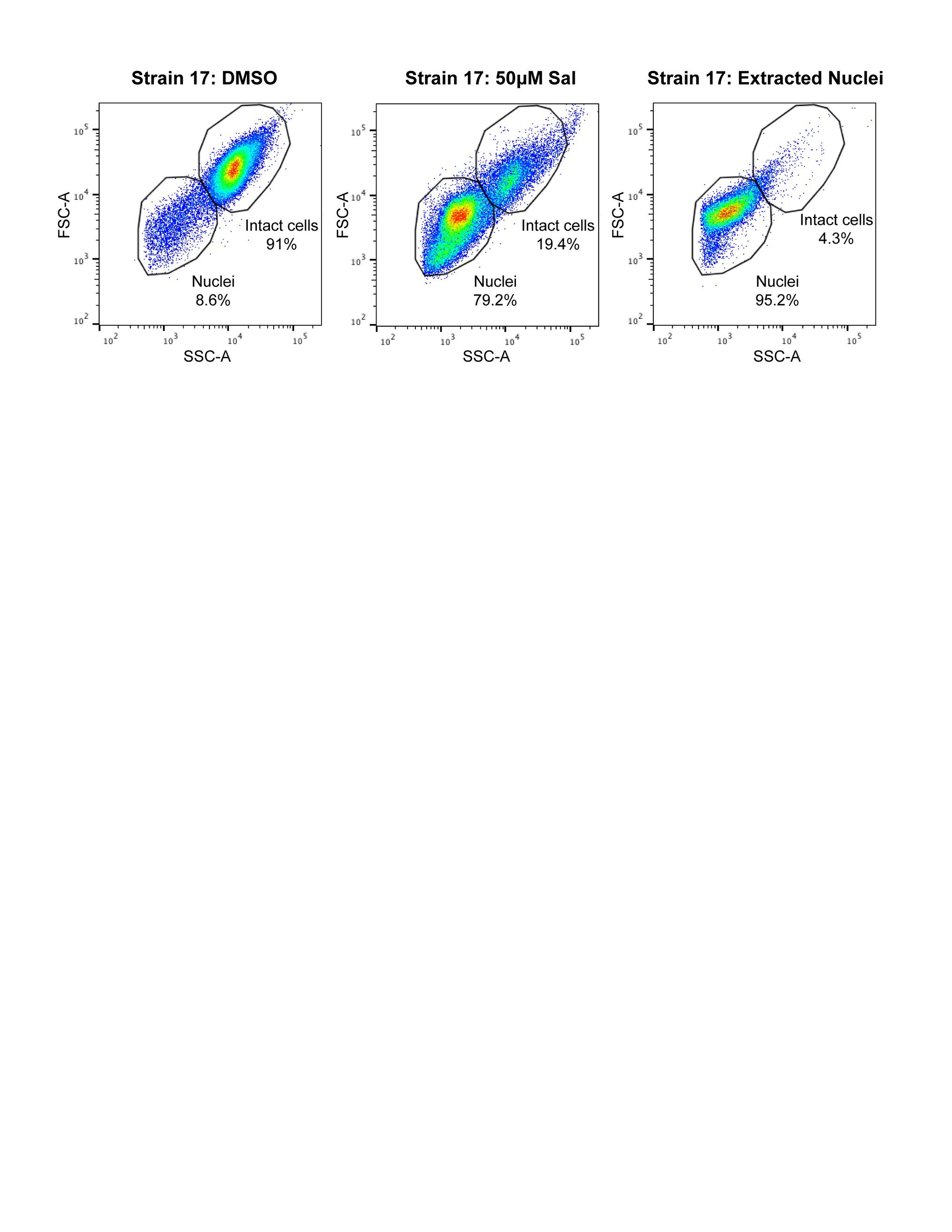

Supplement: S1 Fig — Samples from strain 17-infected cells were prepared and run through a BD LSRFortessa cell analyzer as described in (Fig 2A and 2B). These dot plots represent the PI-positive events as measured by SSC-A and FSC-A. The intact cell population and free nuclei population are denoted by the drawn circles and accompanying percentages. (TIF) [file ppat.1007054.s001.tif]

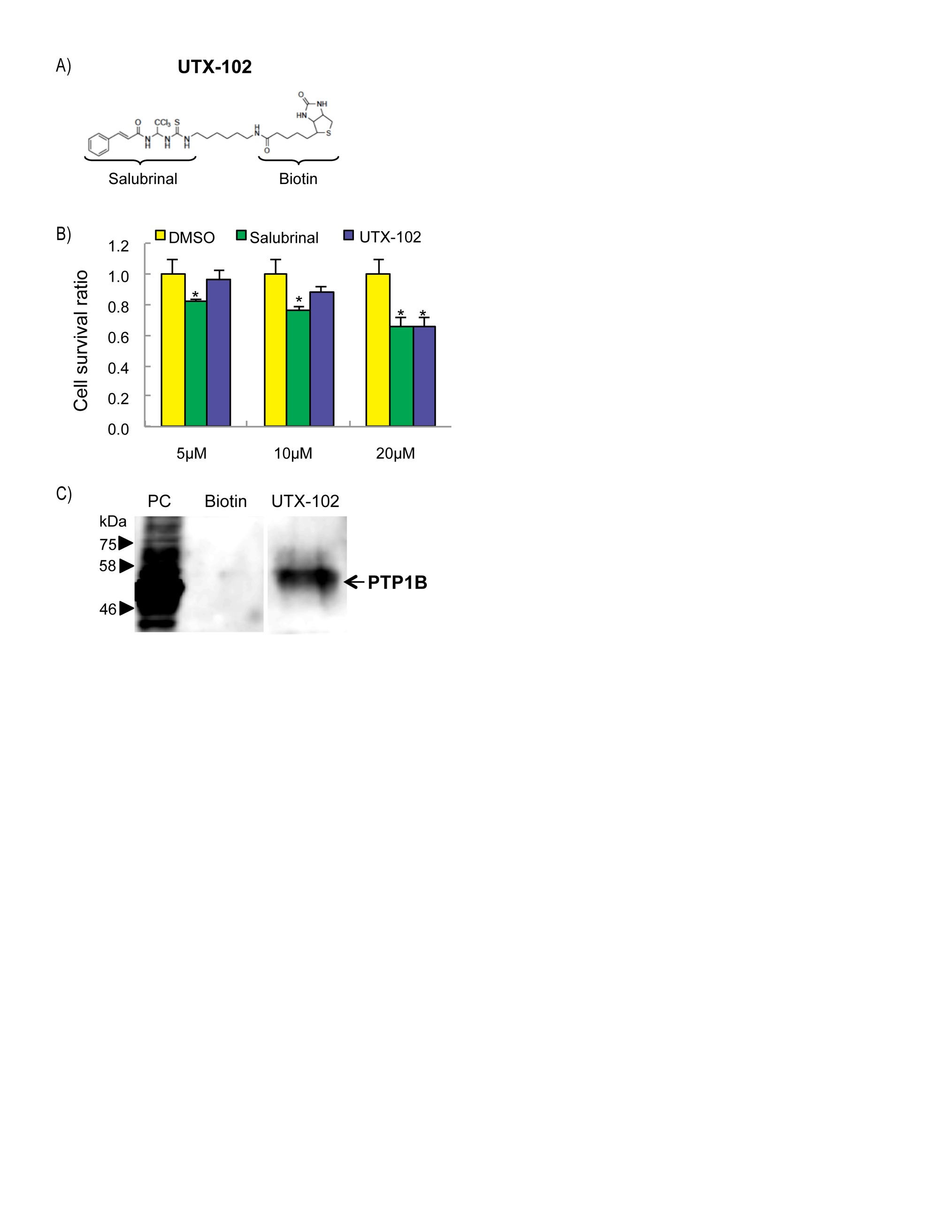

Supplement: S2 Fig — (A) Diagram of salubrinal analogue, UTX-102. Segments representing salubrinal and biotin are indicated. (B) MDA-MB-231 cells were treated with DMSO, salubrinal, or UTX-102 in increasing amounts. Cell viability was measured and the cell survival ratio calculated. (C) MDA-MB-231 cells were treated with 20 μM UTX-102 for 5 hours, and total cell proteins were harvested in the presence of phosphatase and protease inhibitors. An avidin-biotin pull-down assay was utilized, and PTP1B was shown to be present in the complex by western blot analysis. Input is listed as PC (protein control) and a biotin-only control was also utilized. (TIF) [file ppat.1007054.s002.tif]

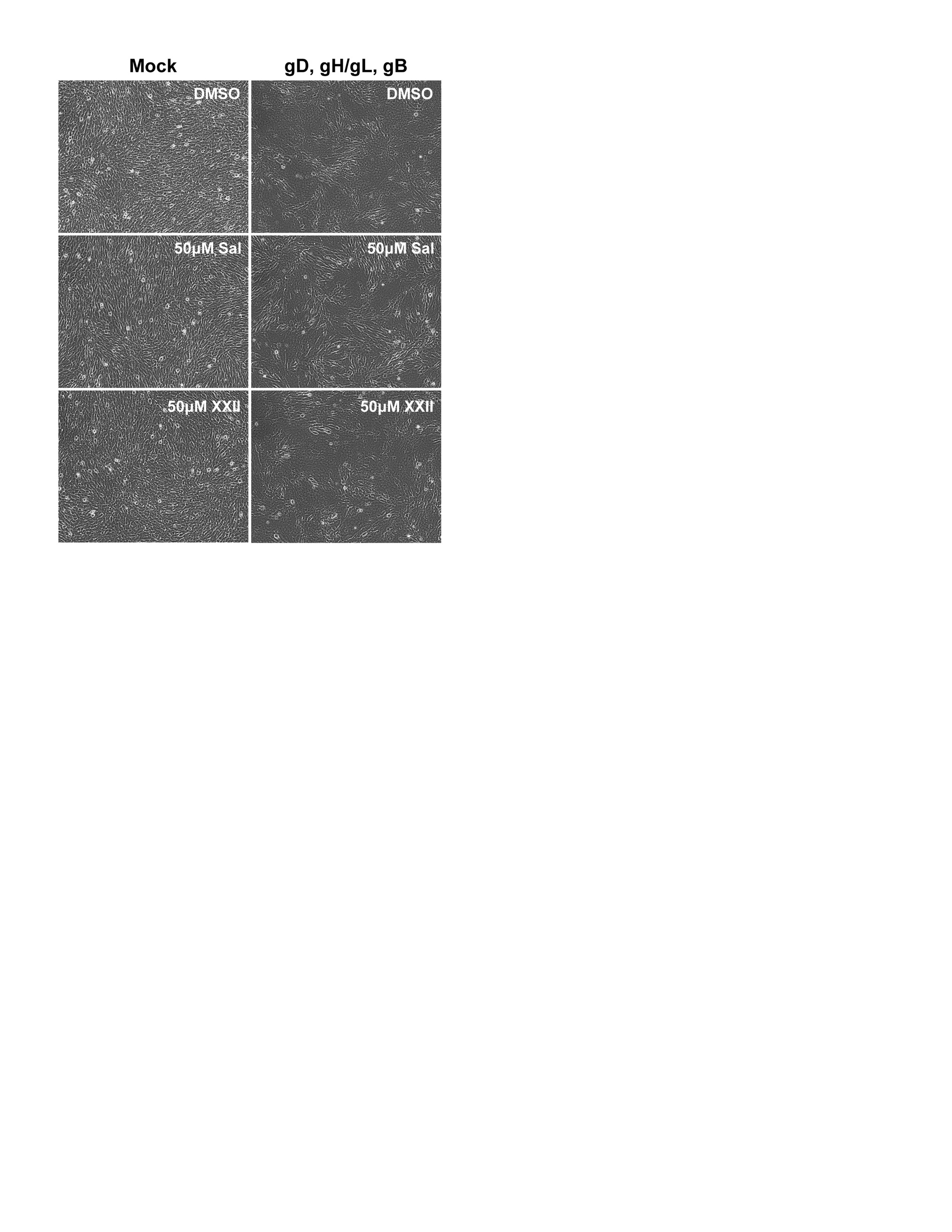

Supplement: S3 Fig — C10 cells were transfected with the core fusion plasmids (gB, gD. gH/gL) in a 3:1:1:1 ratio. Transfected cells were treated with DMSO, 50 μM salubrinal, or 50 μM inhibitor XXII. These images were taken at 24 hours post transfection. (TIF) [file ppat.1007054.s003.tif]

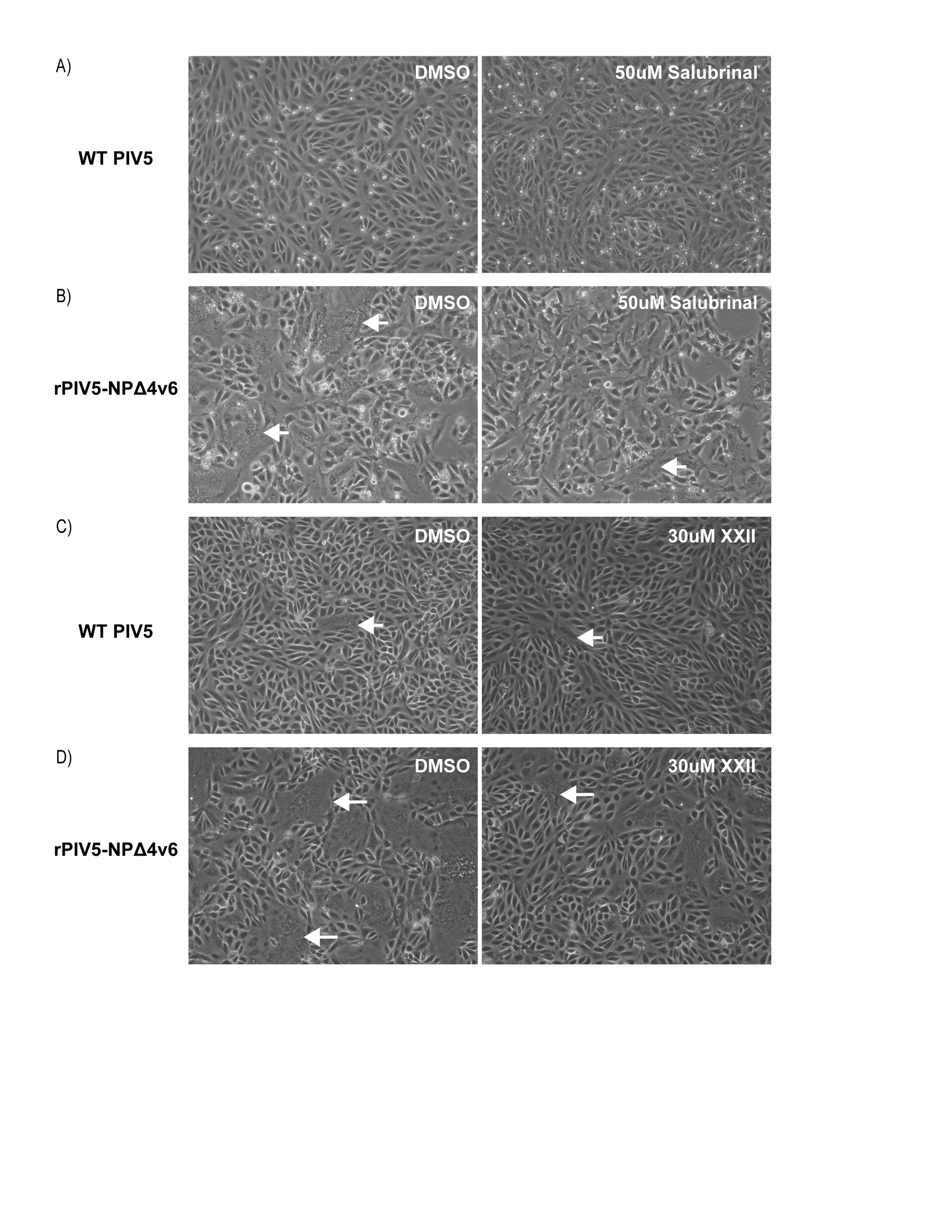

Supplement: S4 Fig — (A and C) Vero cells were infected with wild-type PIV5 at an MOI of 1 and incubated in medium containing DMSO, 50 μM salubrinal, or 30 μM inhibitor XXII, as indicated. Images were taken 96 hpi. Examples of syncytia are indicated with arrows. (B and D) Vero cells were infected with fusogenic mutant rPIV5-NPΔ4v6 at an MOI of 0.1 and incubated with DMSO, 50 μM salubrinal, or 30 μM inhibitor XXII, as indicated. Images were taken at 24 hpi. (TIF) [file ppat.1007054.s004.tif]

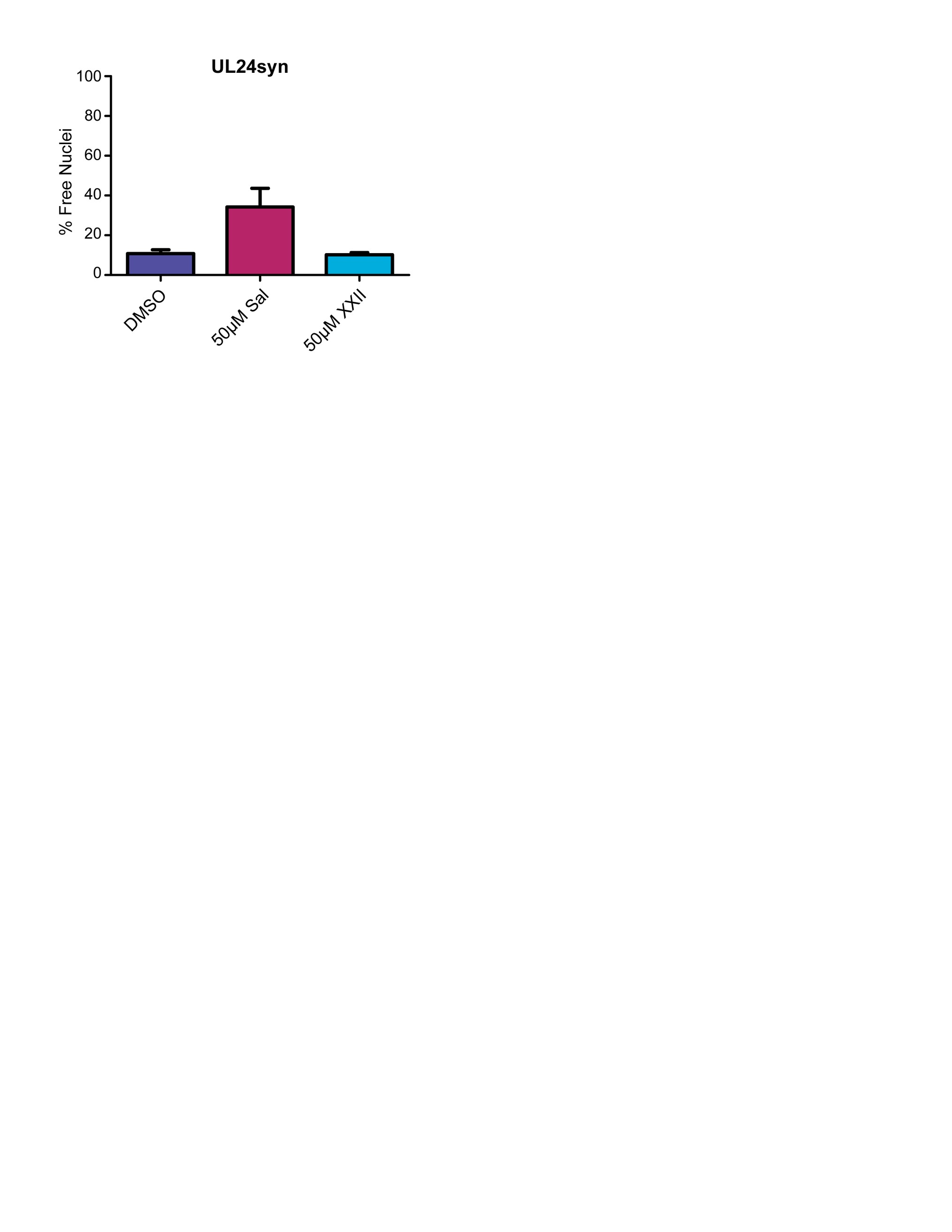

Supplement: S5 Fig — Vero cells were infected with Syn mutant UL24.G121A at an MOI of 3 and incubated with DMSO, 50 μM salubrinal, or 50 μM inhibitor XXII. At 12 hpi, the cells were harvested and fusion was assessed by flow cytometry. The averages from two independent experiments are shown. (TIF) [file ppat.1007054.s005.tif]

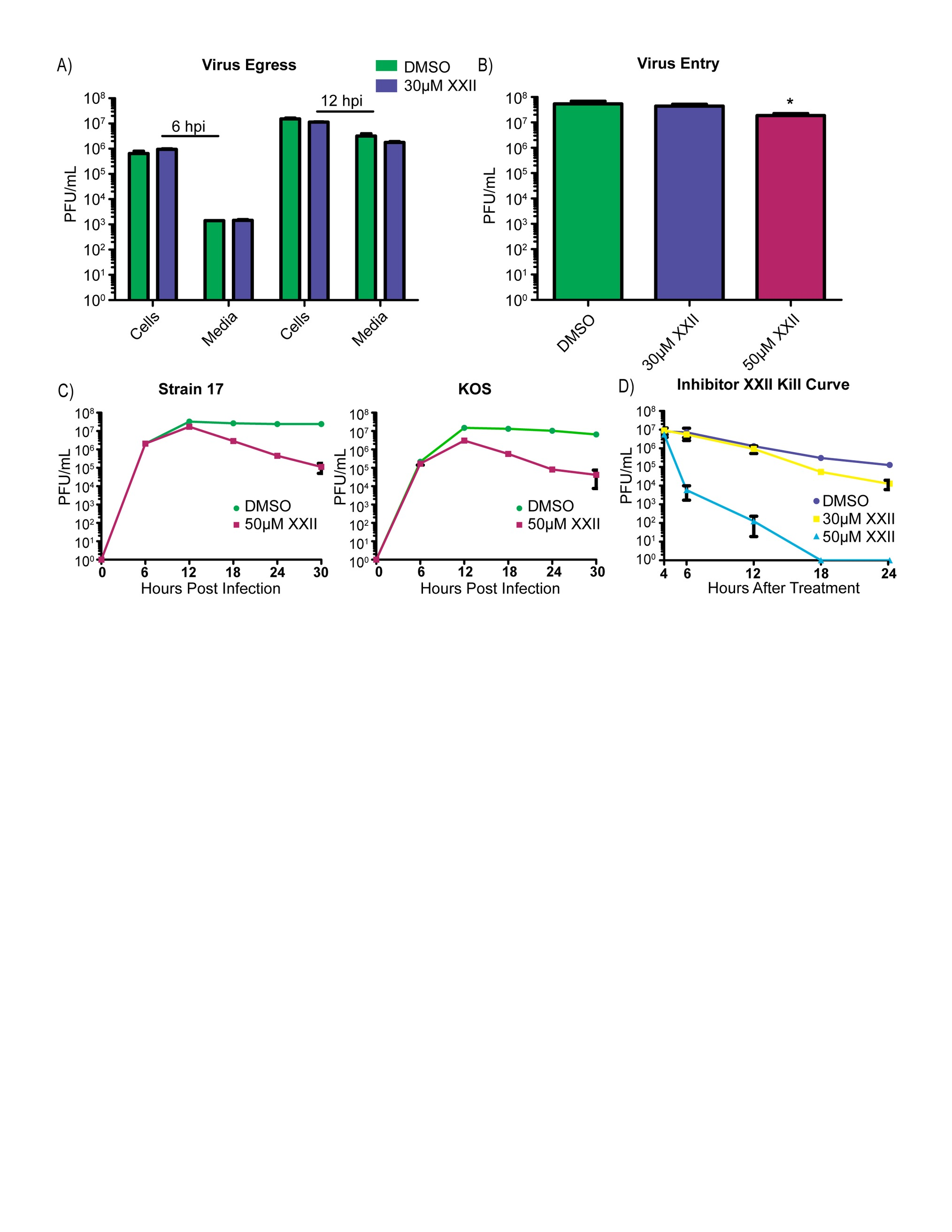

Supplement: S6 Fig — (A) Vero cells were infected with strain 17 (MOI = 5) and treated with DMSO or 30 μM inhibitor XXII. At 6 and 12 hpi, infected cell lysates and media were harvested separately, and the virus titers were measured for each. (B) Vero cells were treated with DMSO, 30 μM inhibitor XXII, or 50 μM inhibitor XXII. After 1 hour of treatment, cells were infected for another hour with serial dilutions of strain 17 in medium also containing DMSO or inhibitor XXII. The cells were then rinsed 2 times and overlaid with methylcellulose for 3 days, and the titers were calculated and represented as mean ±SD from 3 independent experiments. (C) Virus replication assays were performed in Vero cells infected (MOI = 5) with strains KOS or 17, which were incubated in medium containing DMSO or 50 μM inhibitor XXII. At 6-hour time points, duplicate samples were collected to measure the virus titers (cell lysate + medium), which were averaged and plotted. (D) Three identical tubes containing 1x10^7^ pfu/ml of strain 17 received either DMSO, 30 μM inhibitor XXII, or 50 μM inhibitor XXII. The tubes were incubated at 37°C, and duplicate samples were collected at the indicated times. The amount of infectious virus present in each sample was measured by plaque assay, and the duplicate measurements were averaged. (TIF) [file ppat.1007054.s006.tif]

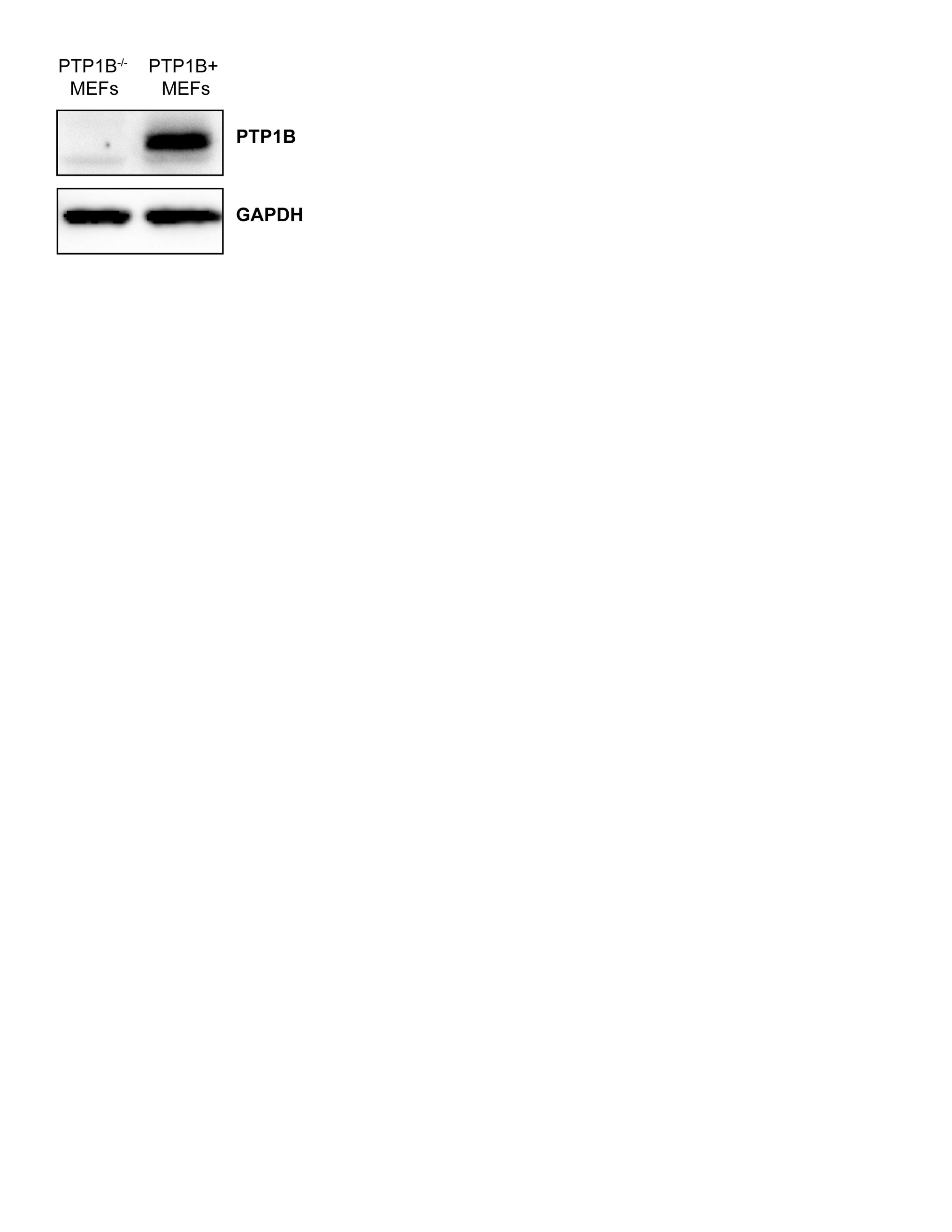

Supplement: S7 Fig — To verify the MEF cell lines used in this study, lysates of PTP1B-/- and PTP1B+ cells were prepared and analyzed by western blotting with PTP1B-specific antiserum. GAPDH was used as a loading control. (TIF) [file ppat.1007054.s007.tif]

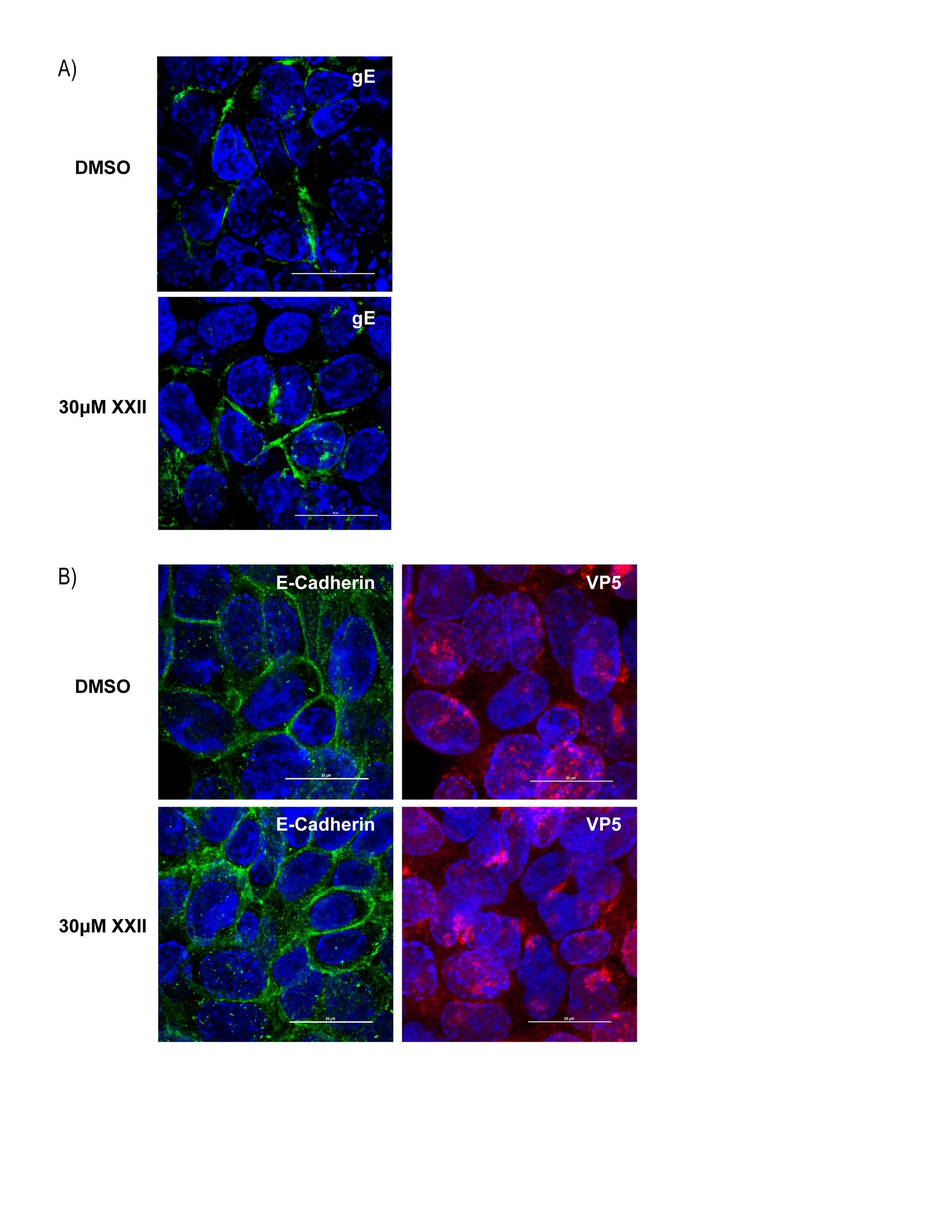

Supplement: S8 Fig — (A) HaCaT cells were infected (MOI = 0.1) with the KOS strain and incubated in the presence of DMSO or 30 μM inhibitor XXII. At 18 hpi, the cells were fixed and immunostained for gE while nuclei were stained with DAPI. Images were taken with a Nikon C2+ confocal microscope, and Z-stacks were collected. Images of representative slices are shown (scale bars indicate 25 μm). (B) HaCaT cells were infected and treated with DMSO or inhibitor XXII as described in (S8A) and were immunostained for E-cadherin or VP5 while nuclei were stained with DAPI. The images for E-cadherin are from one slice of the Z-stack while the VP5 images show the maximum projection of the same Z-stack. (TIF) [file ppat.1007054.s008.tif]

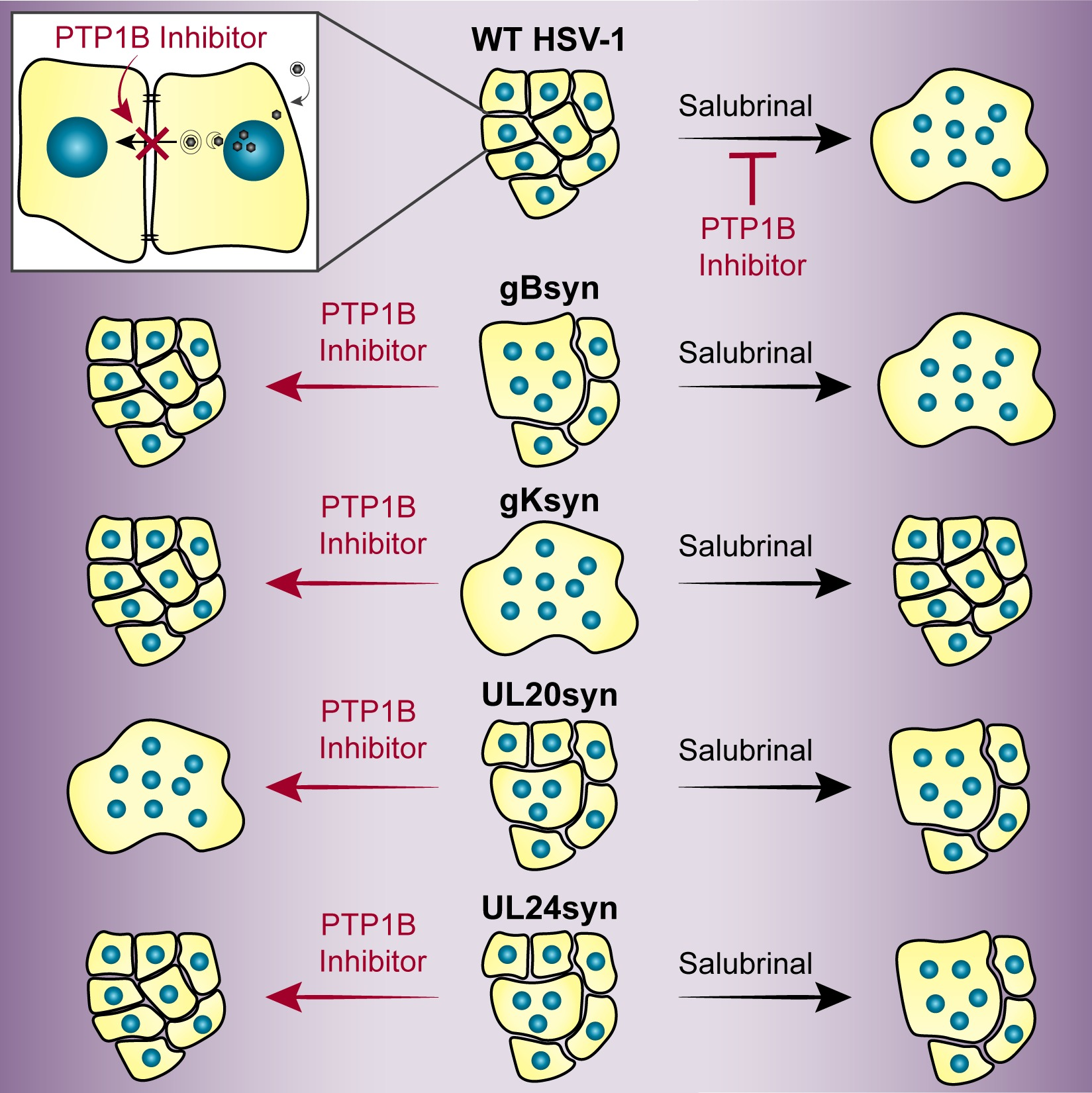

Supplement: S9 Fig — For cells infected with wild-type HSV-1 (top panels), salubrinal stimulates fusion, but this is blocked by the PTP1B inhibitor. By itself, the PTP1B inhibitor blocks cell-to-cell spread. For the four different types of syncytial viruses (remaining panels), the effects of the two drugs depend on which Syn mutant is used. Unexpectedly, salubrinal blocks cell fusion for gKsyn mutants, and inhibition of PTP1B stimulates fusion of cells infected with UL20syn mutants. Collectively, these findings reveal for the first time that tyrosine phosphorylation and dephosphorylation are critical regulators of the viral machinery involved in cell-to-cell spread. Syn mutants dysregulate the machinery and are also critically dependent upon tyrosine phosphorylation and dephosphorylation. (TIF) [file ppat.1007054.s009.tif]
